# KdmA, a histone H3 demethylase with bipartite function, differentially regulates primary and secondary metabolism in *A* *spergillus nidulans*

**DOI:** 10.1111/mmi.12977

**Published:** 2015-04-11

**Authors:** Agnieszka Gacek‐Matthews, Luke M. Noble, Clemens Gruber, Harald Berger, Michael Sulyok, Ana T. Marcos, Joseph Strauss, Alex Andrianopoulos

**Affiliations:** ^1^ Fungal Genetics and Genomics UnitDepartment of Applied Genetics and Cell BiologyBOKU‐University of Natural Resources and Life SciencesCampus TullnTulln3430Austria; ^2^ Department of GeneticsUniversity of MelbourneVictoria3010Australia; ^3^ Department of ChemistryBOKU‐University of Natural Resources and Life SciencesCampus MuthgasseViennaA‐1190Austria; ^4^ Health and Environment DepartmentAIT – Austrian Institute of Technology GmbHCampus TullnTulln3430Austria; ^5^ Center for Analytical ChemistryDepartment IFA TullnBOKU‐University of Natural Resources and Life SciencesCampus TullnTulln3430Austria; ^6^ Departamento de GenéticaFacultad de BiologíaUniversidad de SevillaSevilla41012Spain

## Abstract

*A* *spergillus nidulans kdmA* encodes a member of the KDM4 family of jumonji histone demethylase proteins, highly similar to metazoan orthologues both within functional domains and in domain architecture. This family of proteins exhibits demethylase activity towards lysines 9 and 36 of histone H3 and plays a prominent role in gene expression and chromosome structure in many species. Mass spectrometry mapping of *A* *. nidulans* histones revealed that around 3% of bulk histone H3 carried trimethylated H3K9 (H3K9me3) but more than 90% of histones carried either H3K36me2 or H3K36me3. KdmA functions as H3K36me3 demethylase and has roles in transcriptional regulation. Genetic manipulation of KdmA levels is tolerated without obvious effect in most conditions, but strong phenotypes are evident under various conditions of stress. Transcriptome analysis revealed that – in submerged early and late cultures – between 25% and 30% of the genome is under KdmA influence respectively. Transcriptional imbalance in the *kdm* *A* deletion mutant may contribute to the lethal phenotype observed upon exposure of mutant cells to low‐density visible light on solid medium. Although KdmA acts as transcriptional co‐repressor of primary metabolism genes, it is required for full expression of several genes involved in biosynthesis of secondary metabolites.

## Introduction

Chromatin modification is widely recognised as an important mechanism in gene and genome regulation, conferring stability and flexibility to expression patterns, cellular memory and positional controls. The core packaging proteins, histones, are subject to a wide variety of regulatory covalent modifications, most commonly involving attachment of methyl‐, acetyl‐, phosphate‐ or ubiquitin groups to particular amino acid residues of histone N‐termini at the nucleosome exterior.

These histone modifications are dynamic and, together with DNA methylation, largely determine chromatin state, that is transcriptionally competent euchromatin or transcriptionally repressed, silent heterochromatin. Of the plethora of known histone modifications, one of the best understood markers for heterochromatic genes is histone H3 lysine 9 methylation (H3K9me2/3). It is a canonical marker of compact, silent, heterochromatic domains (Tschiersch *et al*., 1994; Allshire *et al*., 1995; Ekwall *et al*., 1995; 1996; Ivanova *et al*., 1998; Eissenberg and Elgin, 2000; Bannister *et al*., 2001; Nakayama *et al*., 2001). Recently, we and other groups showed that in filamentous saprophytic and pathogenic fungi gene clusters coding for low molecular weight compounds such as toxins, antimicrobial substances or virulence factors are de‐repressed upon depletion of elements required for heterochromatin formation (Bok *et al*., 2009a; Reyes‐Dominguez *et al*., 2010; Boedi *et al*., 2012; Soyer *et al*., 2014). However, in *Aspergillus nidulans*, amino acid substitution of histone H3 lysine K9 to arginine (H3K9R) does not lead to an altered secondary metabolites (SM) cluster gene expression profile or other obvious phenotypic changes under a range of conditions (Nuetzmann *et al*., 2013). Thus, it is not clear how H3K9me2/3 and HepA [orthologue of heterochromatin protein‐1 (HP1)] binding are involved in SM gene cluster regulation, and it remains to be determined to which extent they directly or indirectly influence the chromatin status and gene expression at the tested loci. Apart from H3K9me2/3, H3K27me3 and the associated chromodomain (CD)‐containing Polycomb Repressive Complex 1 are also associated with heterochromatic regions (Dahiya *et al*., 2001; Heard, 2005; Feil and Berger, 2007; Sessa *et al*., 2007). Recently, genome‐wide distribution of this mark was studied in *Neurospora crassa* (Jamieson *et al*., 2013) and *Fusarium graminearum* (Connolly *et al*., 2013), and in both cases H3K27me3 domains were found in various secondary metabolite gene clusters. Biosynthesis of symbiosis‐specific alkaloids was also shown to be regulated by H3K27me3 – in combination with H3K9me3 and Hep1 – in the plant symbiont *Epichloë festucae* (Chujo and Scott, 2014). Typical features of transcriptionally competent euchromatin are H3K4me2/3 and H3K36me2/3. Both marks form almost mutually exclusive domains within the coding regions of active genes where H3K4me3 is mainly associated with the initiating form of RNA polymerase II (PolII) at the 5′ end of genes, whereas H3K36me3 is associated with the elongating form of PolII and thus is dominant in gene bodies of actively transcribed genes (Liu *et al*., 2005; Liu *et al*., 2011; Roudier *et al*., 2011). In budding yeast, Set‐2 mediated H3K36me3 formation was shown to be involved in control of elongation fidelity and in preventing transcription initiation from cryptic promoters within transcribed genes. This mechanism requires SET2–HDAC interaction maintaining low levels of histone acetylation within gene bodies. In addition, H3K36me3 inhibits histone exchange within the coding region of transcribed genes (Li *et al*., 2002a; Krogan *et al*., 2003; Carrozza *et al*., 2005; Smolle *et al*., 2013). The function and distribution of H3K36me3 in *A. nidulans* is unknown at present. Repeat‐induced point mutation (RIP) of *N. crassa* Set‐2 leads to developmental defects, whereas histone H3K36 exchange to leucine is lethal in this fungus (Adhvaryu *et al*., 2005). The emerging picture of histone modifications in fungi is one of remarkable diversity in function and phenotypic effects, with recent genome wide ChIP‐seq studies in *F. graminearum* finding no association between this mark and active transcription (Connolly *et al*., 2013).

Given the central role of histone methylation in control of gene expression in diverse organisms, and the strong effect on phenotypes in fungi such as secondary metabolism (SM), pathogenicity and endophytic growth, we set out to study the role of histone demethylases in the *A. nidulans* model system beginning with an evolutionarily conserved family of histone demethylases, the jumonji domain genes. The jumonji domain, first identified through a gene trap screen for developmental mutants in mice (Takeuchi *et al*., 1995), refers to N‐terminal (JmjN) and (JmjC) subdomains that are often found together. The JmjC domain, with cofactors Fe^2+^ and α–ketoglutarate, catalyses oxidative demethylation of methyl‐lysine residues (Clissold and Ponting, 2001; Klose *et al*., 2006a; Tsukada *et al*., 2006). JmjC proteins contain additional domains typically found in transcription factors or chromatin associated proteins such us Bright/Arid, plant homeodomain (PHD), C_2_H_2_ zinc finger or Tudor (Clissold and Ponting, 2001). Domain composition of JmjC containing proteins and the architecture of a catalytic domain affects their substrate specificity and is the basis for family and subfamily classification. We generated deletion strains for a number of JmjC domain genes and here we report the characterisation of the first one, named *kdmA*, encoding an orthologue of metazoan H3K36me2/3 and H3K9me2/3 demethylase, a member of JHDM3/JMJD2 family (Chen *et al*., 2006; Whetstine *et al*., 2006) now termed KDM4 family under the unified nomenclature (Allis *et al*., 2007). Until now, KDM4 family demethylases from yeast to animals have been shown to be involved in the regulation of metabolic activity and development. Mammalian JMJD2A antagonizes H3K9me3 and H3K36me3 at euchromatic or facultative heterochromatic loci contributing to transcriptional repression of JMJD2A targeted genes (Klose *et al*., 2006b). Overexpression of *Saccharomyces cerevisiae* H3K36me3 demethylase Rph1 leads to growth defects, but this function is not dependent on enzymatic activity (Klose *et al*., 2007a) suggesting that Rph1 functions as a classical DNA‐binding transcriptional repressor in this case. An estimated 75% of Rph1 targeted genes (containing putative binding sites) are induced upon *RPH1* deletion, revealing the direct repressor function of this protein. Genes responding to *RPH1* knockout are mainly involved in environmental stress response, normally induced by oxidative stress or DNA damage. In this system, Rph1‐mediated repression is released after Rph1 dissociation from the promoter, allowing the binding of an appropriate activator (Liang *et al*., 2013).

We found in our study that KdmA is a H3K36 demethylase with roles in transcriptional regulation. Deletion of the gene detectably alters transcription of around 30% of the *A. nidulans* genome during different metabolic phases. KdmA is necessary to respond to photo stimuli for growth and development and to tolerate the detrimental effects of light, making a mutant highly light‐sensitive. The deletion mutant shows a gene–environment interaction with hypoxia, a key cue for sexual development. We show that genes associated with energy metabolism, protein production and stability are upregulated in the mutant, suggesting a negative role for KdmA in these metabolic processes (directly or indirectly). In contrast, some genes transcribed during SM have strongly reduced transcript levels in the mutant and thus for some of these genes KdmA may act as a facilitator/coactivator of transcription possibly by counteracting repressive heterochromatic structures. Thus, the bipartite function of this histone H3 demethylase seems to be related to the metabolic and developmental phase of *A. nidulans* cells.

## Results

### Predicted JmjC domain lysine demethylases in *A* *. nidulans*

**Figure 1:**
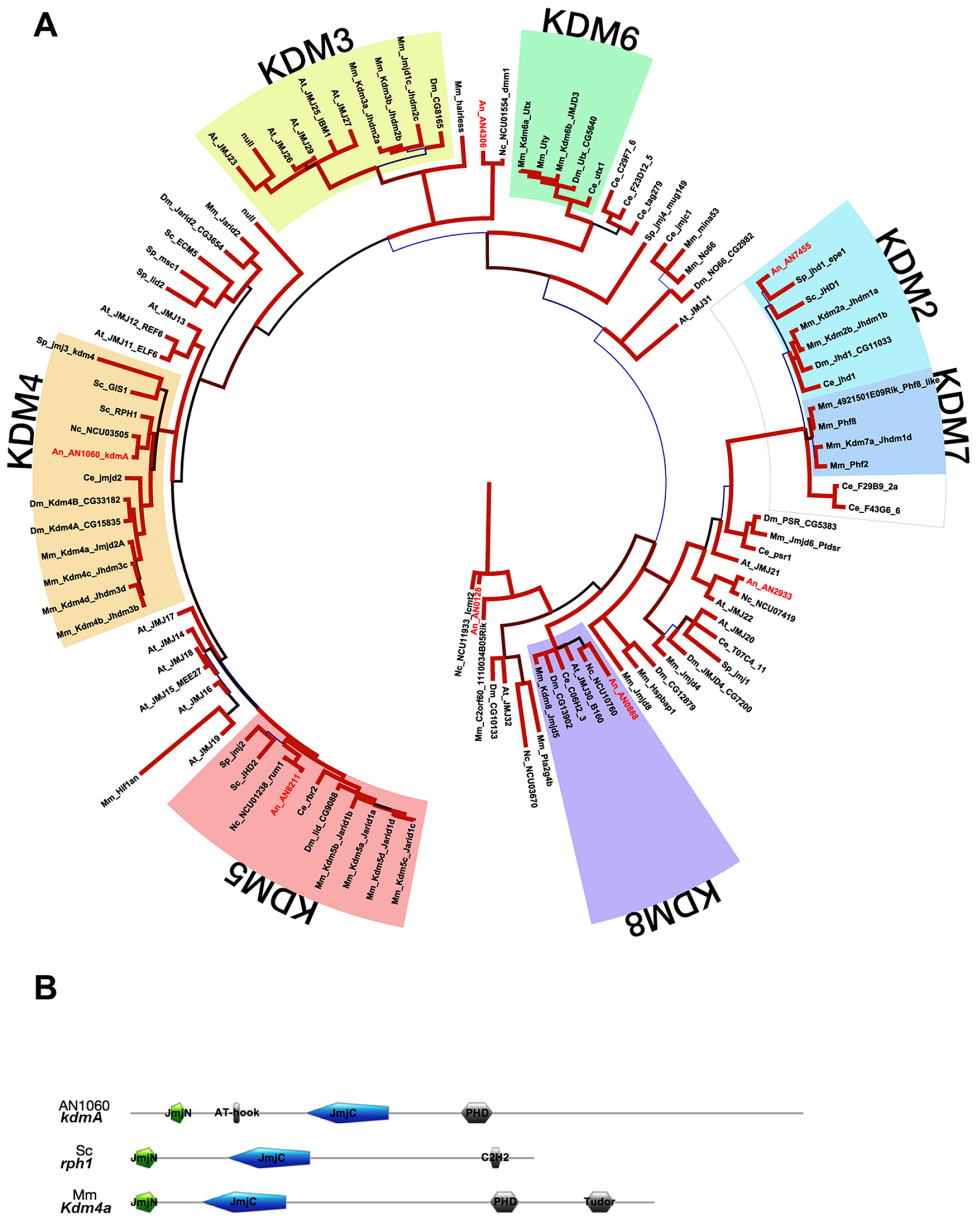
JmjC domain protein phylogeny across fungal, nematode, *D* *rosophila*, plant and vertebrate model organisms. A. JmjC protein domain (Interpro IPR003347) phylogeny. Domains were aligned by Clustal multiple sequence alignment and analysed by Bayesian inference of phylogeny. JmjC domain KDM families (KDM2‐8) are highlighted and *A. nidulans* proteins are shown in red. Species represented are *Saccharomyces cerevisiae* (Sc), *Schizosaccharomyces pombe* (Sp), *Aspergillus nidulans* (An), *Neurospora crassa* (Nc), *Caenorhabditis elegans* (Ce), *Drosophila melanogaster* (Dm), *Arabidopsis thaliana* (At) and *Mus musculus* (Mm). Branch colours (gradient from bright red = 95–100%, through black = 70–80%, to light blue = 50–60%) and widths (5 point gradient) represent posterior probability approximations. B. Domain composition of JHDM3/JMJD2 histone demethylases. Mammalian Kdm4a targets H3K9,36me2/3, *S. cerevisiae* RPH1 lacking both Tudor and PHD domains, removes H3K36me3 and *in vitro* H3K9me3 mark. *A. nidulans* orthologue KdmA contain DNA and protein binding domains but lack the Tudor domain.

To survey potential JmjC domain histone demethylases in *A. nidulans*, phylogenetic analysis from model organisms spanning the plant, animal and fungal kingdoms was conducted based on full‐length proteins and on JmjC domains only (Fig. 1A). Restriction to domains resulted in greater discrimination, but results were not substantially different – established mammalian families were consistently grouped as terminal branches, although we found little sequence differentiation between KDM2 and KDM7 families. Representatives of JmjC domain families found in *A. nidulans* were AN7455 (KDM2), AN1060 (KDM4), AN8211 (KDM5) and AN0888 (KDM8). In addition, AN2933 is similar to mouse jmjd6, originally designated in error as a phosphatidylserine receptor and yet to receive a systematic name under the unified nomenclature (Allis *et al*., 2007), and AN4306 is similar to *N. crassa* dmm‐1, which is required for restriction of H3K9me to silenced transposable elements (Honda *et al*., 2010). Clearly distinct from other JmjC domain proteins is AN0128, which lacks other motifs implicated in DNA or chromatin interaction such as various zinc finger or AT‐rich interaction domains (Fig. 1B). AN0128 encodes leucine carboxyl methyltransferase and galactose oxidase domains, consistent with the function of its *S. cerevisiae* orthologue, Ppm2, as a mitochondrial protein involved in hydroxylation of the hypermodified nucleoside wybutosine (Noma *et al*., 2006; Iyer *et al*., 2010). Although AN0888 also lacks other domains characteristic of chromatin modification proteins, its JmjC domain clusters closely within the KDM8 family, the mouse member of which possesses H3K36me demethylase activity (Ishimura *et al*., 2012; Oh and Janknecht, 2012).

AN1060 (designated as *kdmA*) is a member of the mammalian KDM4 family of proteins (also known as JHDM3/JMJD2 in mammals), containing *S. cerevisiae* paralogues *RPH1* and *GIS1* (Allis *et al*., 2007). All characterised proteins within this family are able to demethylate di‐ and trimethylated lysine, including lysines 9 (although *S. cerevisiae* does not feature H3K9 methylation) and 36 of histone H3 (Cloos *et al*., 2006; Kim and Buratowski, 2007; Klose *et al*., 2007b; Sun and Zhou, 2008; Ponnaluri *et al*., 2009). Both the JmjC domain and general domain architecture of *kdmA* are similar to orthologues from studied species, including a chromatin binding PHD zinc finger, an N‐terminal JmjN domain and two AT‐hooks (Fig. 1B). JmjC domain similarity is 50–55% for mouse kdm4 members and 63% for *S. cerevisiae* Rph1 and includes strictly conserved residues required for binding cofactors Fe(II) and α‐ketoglutarate (Chang *et al*., 2010). Notably, the tandem Tudor domains known to recognise methylated H3K4, H3K9 or H4K20 in chromatin and present in the mammalian isoforms JMJD2A, B and C are missing in KdmA. Chromatin binding of KdmA could be mediated instead by the predicted C_4_‐H‐C_3_ PHD Zn finger structure which was shown to interact with H3K9me3 in the JARID1C demethylase (Iwase *et al*., 2007) and with H3K4me0 (unmethylated lysine 4 in H3) in BHC80, a subunit of the LSD1 demethylase complex (Lan *et al*., 2007).

### MS/MS mapping of Histone H3 methylation

**Figure 2:**
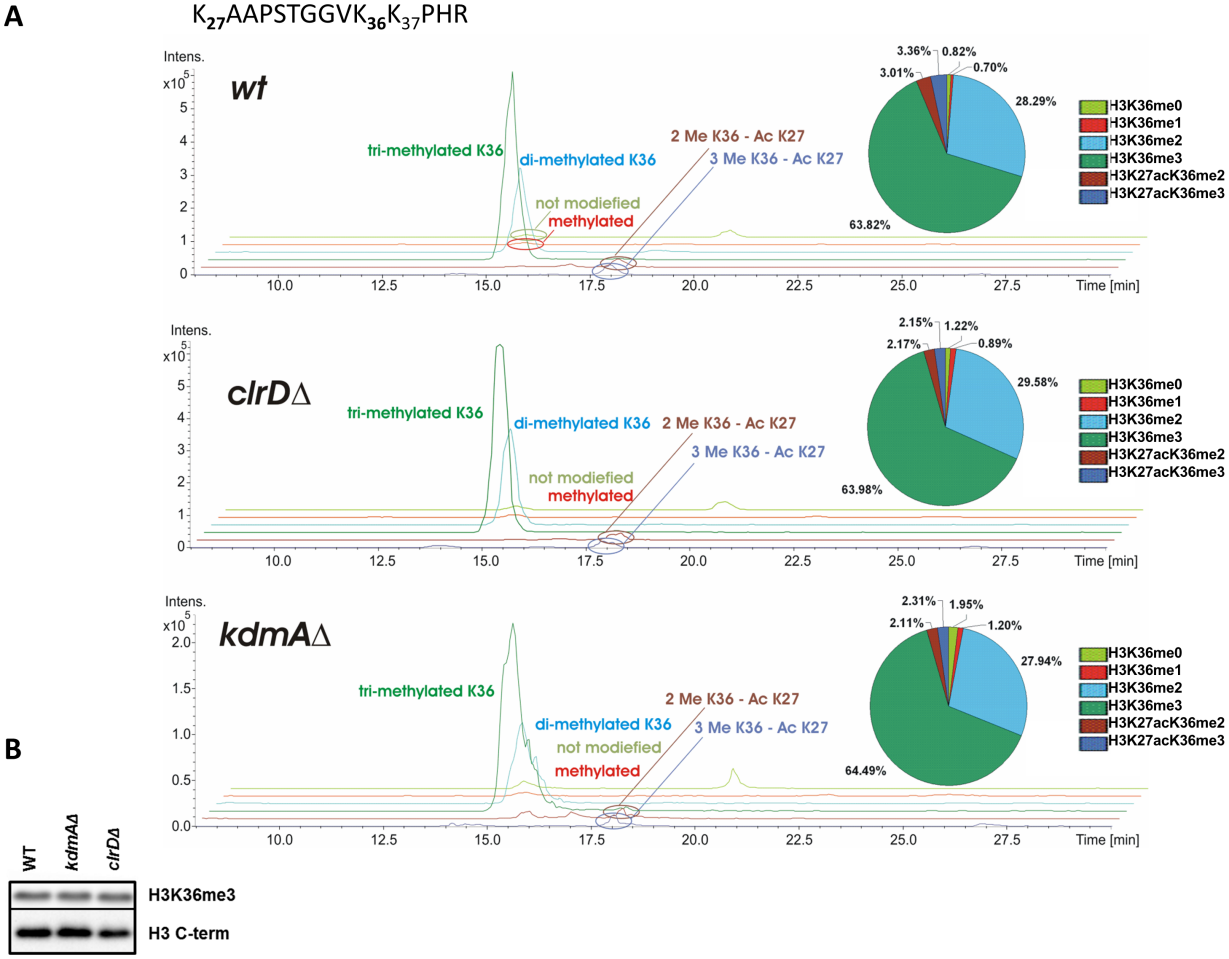
LC‐MS/MS determined *H* *3* *K* *36me3 levels in* *WT* *, kdm* *A* *Δ and clr* *D* *Δ.* **A.** LC‐MS/MS Base Peak Chromatograms (BPC) and ratios of differently modified variants of the histone H3 K_27_AAPSTGGVK_36_K_37_PHR peptide from WT, *clrDΔ* and *kdmAΔ*. **B.** Western blot with antibody specific to H3K36me3 and histone H3‐ C term. 15 µg of acidic extracted *A. nidulans* histones were loaded.

So far, a systematic biochemical analysis of histone post‐translational modifications present in *A. nidulans* has not been carried out and we thus performed mass spectrometry (MS) to determine the extent of methylation at histone H3 lysine 9 and lysine 36, the putative targets of KdmA. LC‐MS/MS analysis of histone H3 obtained from acidic nuclear extracts prepared from wild type, *kdmAΔ* and *clrDΔ* cells (devoid of H3K9 methylation activity) grown for 17 h [active growth, primary metabolism (PM)] revealed peptides (K_27_AAPSTGGVK_36_K_37_PHR) in which di‐ and trimethylated K36 was dominant: 92% of peptides carried either of the two marks, around 28% of the peptides carried H3K36me2 and around 64% carried H3K36me3. In contrast, mono‐methylated and unmodified K36 together represented only around 1.5% of the peptides. The remaining fraction was represented by peptides that carried, in addition to the H3K36 methylation marks, an acetylation mark on K27 (Fig. 2A). Acetylation of H3K27 occurred only when H3K36 was di‐ or trimethylated indicating a co‐transcriptional effect of this mark. Strikingly, methylation of H3K27 – a crucial modification associated with repressed chromatin in other fungi – was not detected in our samples. This is consistent with a lack of genes in the *A. nidulans* genome predicted to code for members of Polycomb Repressive Complex 2 known to methylate H3K27 in mammals and other fungi.

We also tested acidic extracts from the *kdmAΔ* strain for the presence of the relevant modification. Both MS measurement of histone H3 fractions and Western blots using anti‐H3K36me3 antibody did not reveal any increase in H3K36me3 levels in the bulk histones of *kdmAΔ* (Fig. 2). This finding is not surprising given the fact that approximately 95% of histones already carry either di‐ or trimethylation marks on H3K36 and thus a further increase by lack of KdmA function cannot be expected at the global level (bulk histone preparations) but may occur at specific loci. Such changes can be detected by the more sensitive Chromatin Immunoprcipitation (ChIP) method.

MS/MS analysis of the *clrDΔ* control strain lacking the histone H3 lysine K9 methyltransferase revealed that methylation on the H3 peptides containing K27 and K36 was identical to wild type (WT). This demonstrates that ClrD is not targeting these lysines in H3 in addition to K9 (see below).

**Figure 3:**
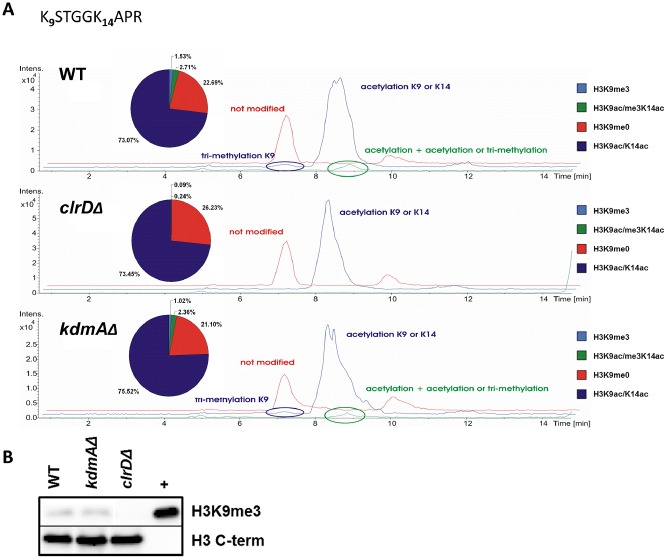
LC‐ MS/MS determined *H* *3* *K* *9me3 levels in* *WT* *, kdm* *A* *Δ and clr* *D* *Δ.* **A**. LC‐MS/MS Base Peak Chromatograms (BPC) and ratios of differently modified variants of the histone H3 K_9_STGGK_14_APR peptide from WT, *clrDΔ* and *kdmAΔ*. **B.** Western blot with antibody specific to H3K9me3 and histone H3‐C term. 15 µg of acidic extracted *A. nidulans* and 2 μg of calf thymus (+) histones were loaded.

As KdmA is predicted to also target lysine 9 at histone H3, we investigated H3 peptides containing this crucial residue. In the K_9_STGGK_14_APR peptide trimethylation of H3K9 was found in roughly 3% of the peptides (between 1.5% and 4% of the analysed K9 peptides; Fig. 3). This H3K9 methylation level of bulk histones is significantly below the levels found in higher eukaryotes and around half the level found in *N. crassa* (Xiong *et al*., 2010). Consistent with the function of ClrD as an H3K9 methyltransferase, H3K9me3 was not detected in *clrDΔ* extracts by neither MS/MS or by Western analysis (Fig. 3). On the other hand, no significant increase in H3K9me3 levels were found by MS/MS or Western blots in H3 peptides of *kdmAΔ* extracts. This suggests that, at the global level, KdmA may not be involved in defining the heterochromatic landscsape. Surprisingly, neither MS measurement nor Western blot analysis using a H3K9me2 specific antibody provided any evidence for the presence of mono‐ or di‐methylated H3K9 in *A. nidulans* (Fig. S1). Latter marks are the dominating marks in fission yeast in which H3K9me3 is also very low (Folco *et al*., 2008). The majority of H3K9 peptides in both WT and mutant were acetylated (around 72%; Fig. 3A) or not modified (around 22%).

We were searching for additional modifications such as arginine methylation or serine phosphorylation, but in this experimental set‐up, no additional modifications on the N‐terminus of H3 were detected. An automated search (MASCOT) as well as manual interpretation of the data were performed to identify differently modified variants of the peptides, obtained from an Arg‐C digestion. Arg‐C is a specific cystein endoproteinase that cleaves at the C‐terminus of arginine residues. Various potential modifications were considered, that is methylation (K,R), dimethylation (K,R), trimethylation (K,R), acetylation (K), phosphorylation (S,T) and oxidation (M), but only those modifications already described in the manuscript were detected (and confirmed by MS/MS). Nevertheless, modifications occurring in marginal proportions may be below the detection limit. Especially modifications of arginine could result in partially missed cleaved variants and thus signal loss or splitting. To reveal methylated arginines, a different set of proteases needs to be employed because the actual enzyme may not efficiently cut the methylated form in the arginine‐lysine combination.

### KdmA displays locus‐specific histone H3 lysine demethylation activity

**Figure 4:**
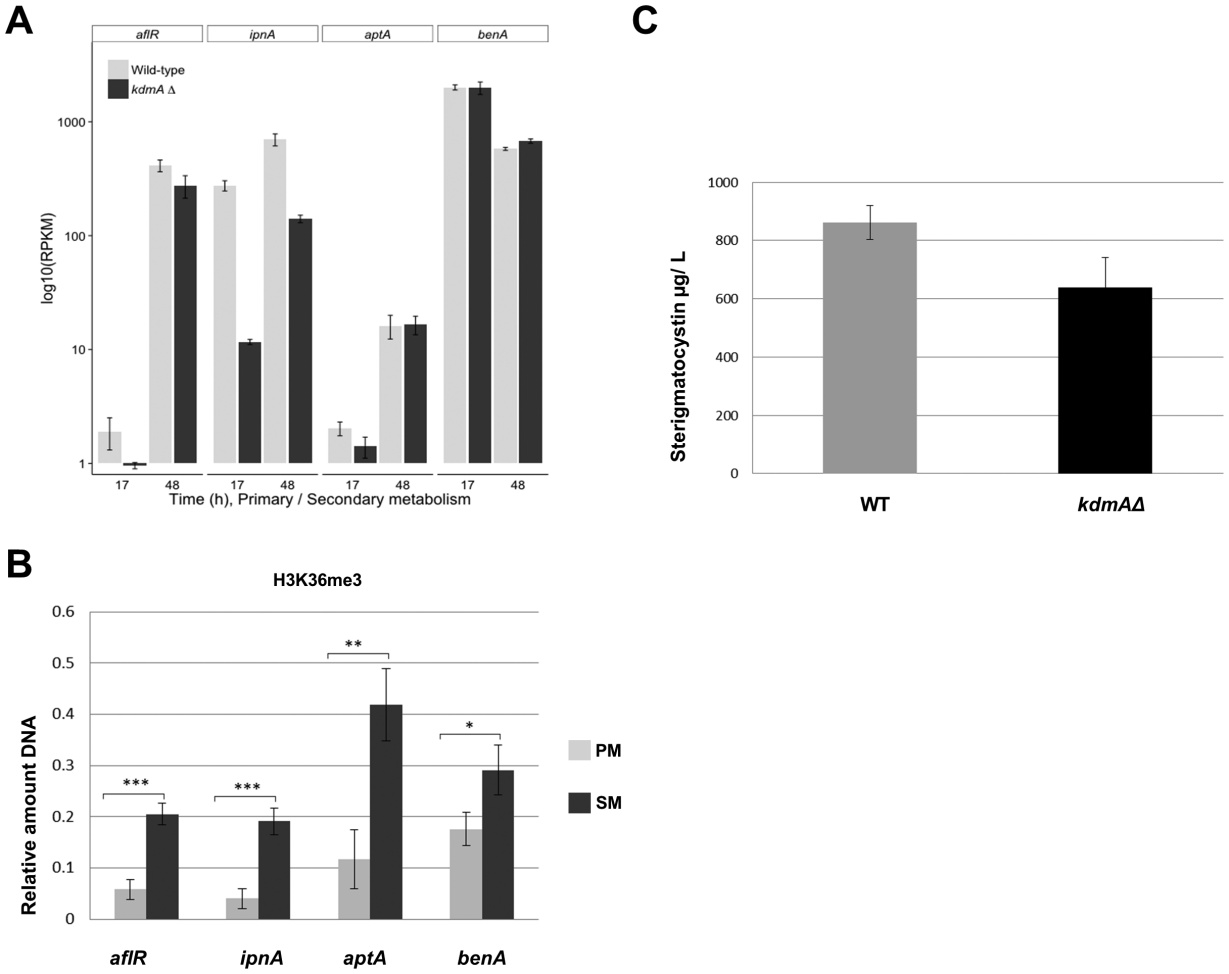
Locus specific H3K36me3 levels increase concomitantly with transcript levels and SM titers. **A.** Expression levels of *aflR*, *ipnA*, *aptA* and housekeeping gene *benA* in 17 h (PM) and 48 h (SM) cultures. Bar chart symbolises normalised read counts of WT and *kdmAΔ.* Standard bars represent standard deviation of two independent biological experiments. **B.** H3K36me3 levels at 3′ regions of *aflR*, *ipnA*, *aptA* and housekeeping gene *benA* detected by qChIP in relation to input DNA. Chromatin was isolated from WT 17 h (PM) and 48 h (SM) liquid cultures. ***P < 0.00005, **P < 0.001, *P < 0.01 determined by *t*‐test: Two‐Sample Assuming Equal Variances. Error bars indicate the standard deviation of at least two biological and two technical replicates. **C.** Sterigmatocystin concentration after 48 h liquid submerged culture in WT and *kdmAΔ*. Cultures were inoculated with defined spore concentration and incubated for 48 h, followed by HPLC‐MS/MS analysis of the supernatants.

As we did not find any significant differences in global histone H3 methylation between wild type and *kdmA* mutants, we performed ChIP analysis to quantify possible locus‐specific differences between the strains. For this, we chose a housekeeping gene (*benA)* and loci belonging to secondary metabolite gene clusters. *aflR* and *ipnA* were previously shown to be regulated by heterochromatic marks and *aptA* was recently discovered to be a biosynthetic gene required for the production of asperthecin (Szewczyk *et al*., 2008). These clusters are silent during PM (17 h standard batch growth conditions on 1% glucose and 10 mM nitrate) and activated during the transition to SM (in our experimental conditions 48 h of growth on the same culture medium). Therefore, for simplicity we call 17 h cultures PM and 48 h cultures SM. Figure 4A shows the transcriptional levels of the genes targeted for chromatin analysis by ChIP based on RNAseq (see below). Under these experimental conditions, *benA* expression is strong and constitutive; *aflR* is induced from very low to high levels whereas *aptA* is only slightly induced in our conditions. In contrast to *aflR* and *aptA*, which are basically silent during active growth (PM), *ipnA* is already transcribed to a significant level in these 17 h cultures.

**Figure 5:**
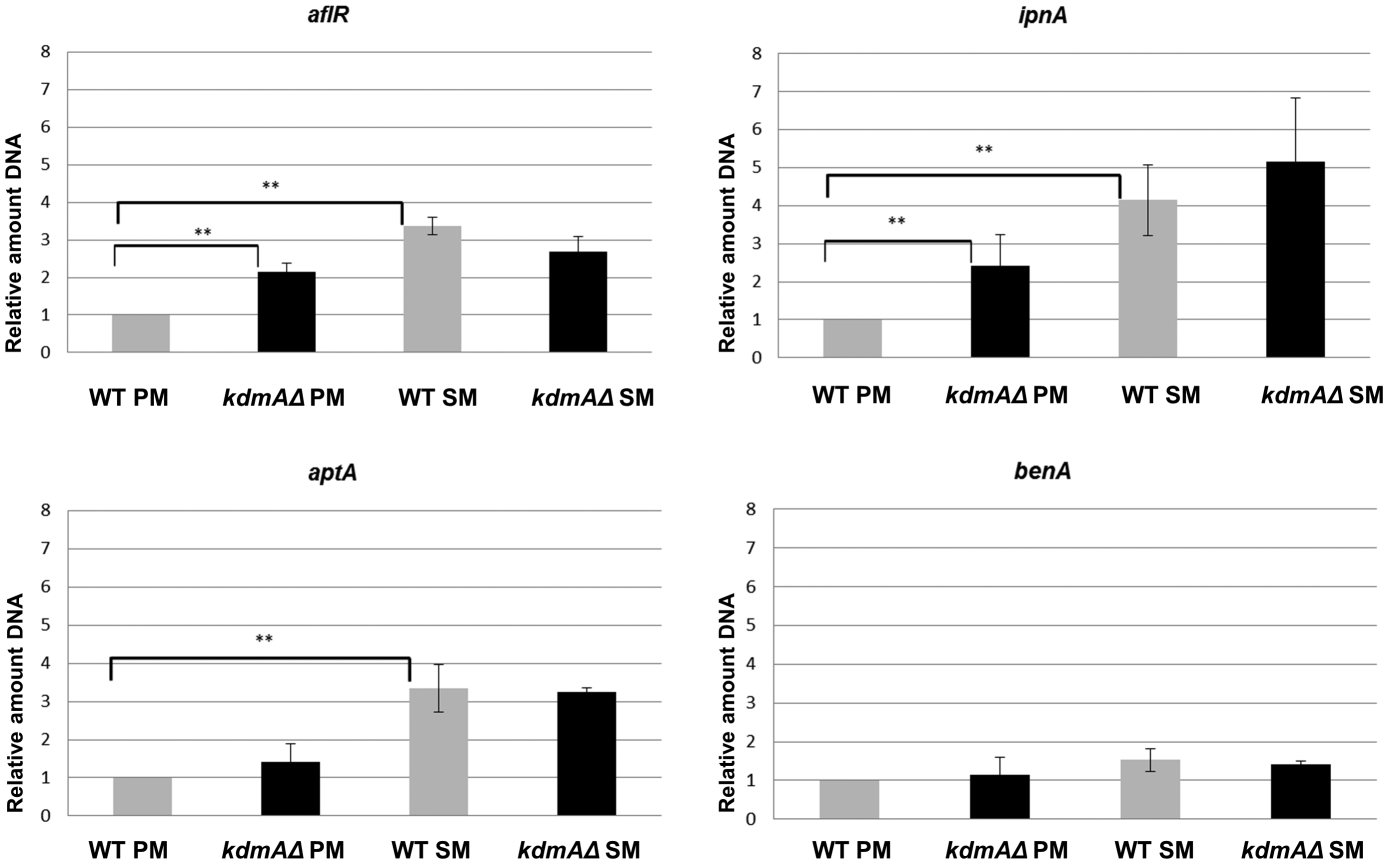
H3K36me3 levels are not necessarily directly correlated to transcriptional activity. ChIP with H3K36me3 antibody followed by qPCR with primers specific to 3′ region of indicated genes. Data in relation to input DNA normalised to WT 17 h *** *P* < 0.001, **P < 0.005, *P < 0.01 determined by *t*‐test: Two‐Sample Assuming Equal Variances. Error bars indicate the standard deviation of at least two biological and two technical replicates.

ChIP analysis using an antibody against H3K36me3 (ab9050) produced clear and significant results consistent with the high abundance of this mark found by MS (roughly 65% of peptides carry this mark under PM growth conditions). ChIP data also demonstrated that this methylation mark is highly enriched when the tested genes are activated (Fig. 4A and B) and that KdmA functions as H3K36me3 demethylase *in vivo* (Fig. 5). In the near constitutively transcribed *benA*, we find very little difference (permanently high levels) of H3K36me3 marks under all experimental conditions, whereas in the tested inducible genes (*aflR, ipnA, aptA*) H3K36me3 levels were low under PM conditions (non‐induced state) and increased several‐fold under SM conditions, in parallel with transcriptional induction (Fig. 4B).

For the *kdmA* mutant, we saw a significant increase in H3K36me3 during PM at the *aflR* and *ipnA* locus and some slightly higher levels at the *aptA* genes (Fig. 5). Not surprisingly, there was no further increase of the already high H3K36me3 levels detected during SM conditions in *aflR, ipnA* and *aptA* at these loci. These results suggest that KdmA *in vivo* functions as H3K36me3 demethylase which, at loci involved in SM, removes this mark under conditions of PM and thus may contribute to silencing of SM genes under PM conditions. However, at PM conditions, the higher H3K36me3 levels did not lead to transcriptional activation of the cognate genes. This is consistent with the well‐known requirement for specific transcription factors for the activation event and suggests that H3K36me3 may be one among other signals required for transcriptional activation or efficient elongation.

Inspection of transcriptional activity during SM conditions in the *kdmA*Δ mutant showed that the majority of inducible genes such as *ipnA* or *aflR* show lower mRNA levels in the mutant (Fig. 4A). This is consistent with reduced sterigmatocystin levels found in the mutant strain (Fig. 4C). Thus, it appears that the H3K36me3 (and possibly H3K9me3) demethylation function of KdmA does not follow the ‘simple’ code of being a repressor due to removal of the positively acting H3K36me3 mark, but more complex and locus‐specific functions need be considered (see *Discussion*).

### Expression profiling reveals bipartite functions of KdmA both under PM and SM conditions

To shed more light on the transcription‐related functions of KdmA and the consequences of its deletion, we performed global gene expression analysis by RNA sequencing (RNAseq) of the cultures grown for 17 h (PM conditions) and 48 h (SM conditions). Prior to sequencing, the reverse transcribed RNAs were checked by qPCR for expression of PM and SM indicator genes (*benA* and *aflR*, respectively) to ensure appropriate culture conditions and verify RNAseq results (data not shown).

Deletion of *kdmA* in *A. nidulans* produces both positive and negative changes in transcriptional readouts and the number of affected genes is different under different conditions. Around 25% of all predicted genes (2682 genes) are differentially regulated (log_2_ ≥ 2) between wild type and mutant in early submerged culture cells. Under these conditions, we find 1125 genes higher and 1557 genes lower expressed in the mutant. At a later growth stage and nutrient restriction (SM conditions, 48 h) about 30% of the genes are differentially expressed (3294 genes) between wild type and mutant, but the proportion between up‐ and downregulated genes (1589 up and 1696 down) remains roughly the same. However, the analysis performed in this work cannot distinguish between direct and indirect effects of KdmA function on gene expression.

**Figure 6:**
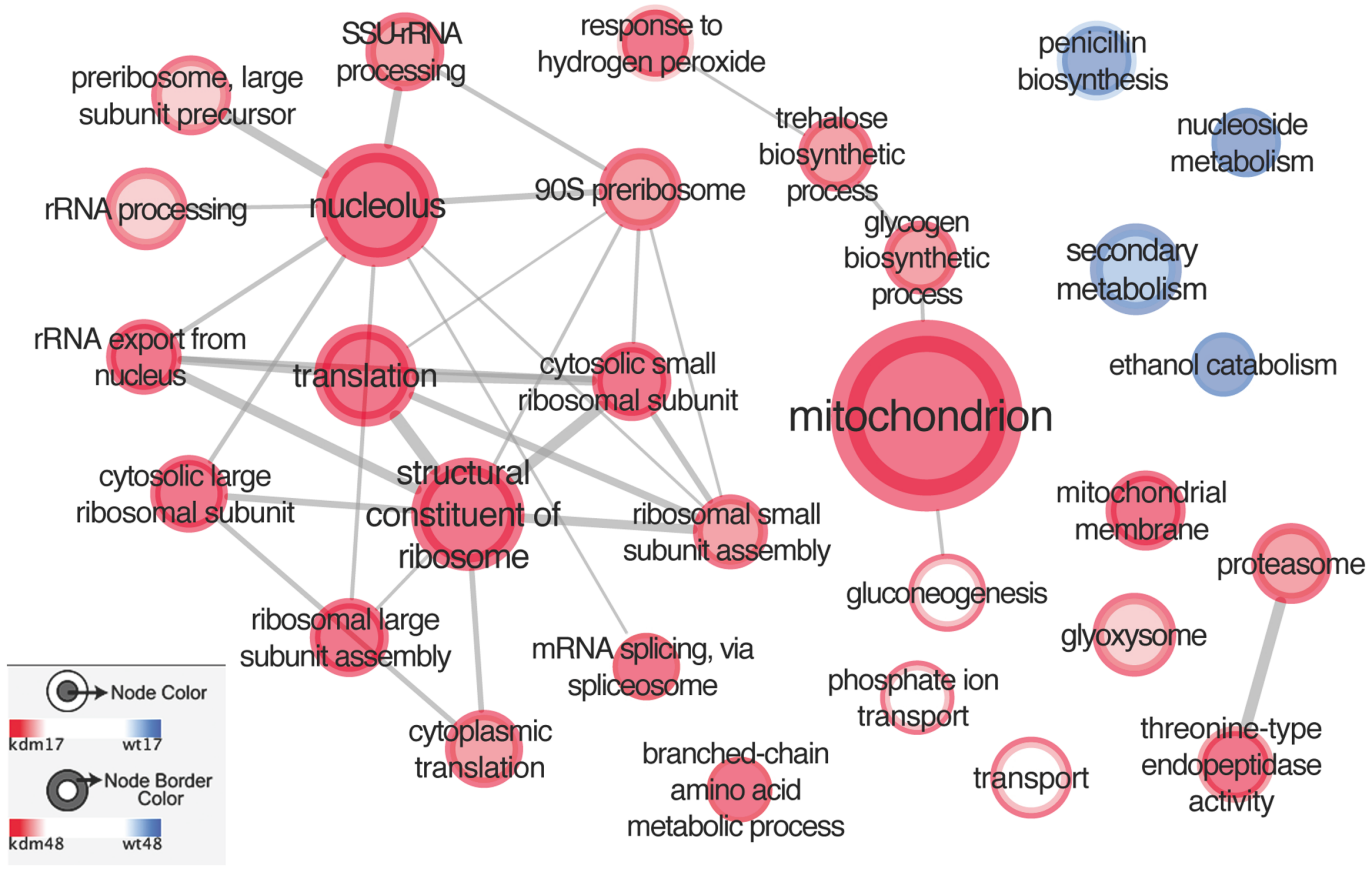
*K* *dm* *A* is a repressor of primary metabolism expressed genes. GO term enrichment network based on gene set enrichment analysis (GSEA) rank results. Node size is scaled by number of genes, node colour by enrichment significance. Aggregates result for *kdm* *A* *Δ*/ WT at 17 h (node centre) and at 48 h (node border). That is, expression of primary metabolism genes is consistently increased in *kdm* *A* *Δ* relative to wild‐type (certain terms such as gluconeogenesis, transport are only significant at 48 h).

Comparison of expression profiles recorded from actively growing wild type and mutant cells (PM conditions) revealed striking differences in the expression patterns. Gene Set Enrichment Analysis (GSEA) shown in Fig. 6 provided evidence that removal of KdmA activity from *A. nidulans* cells results in upregulation of many genes involved in basic metabolism. In our graphical representation of the GSE analysis, the size of the node represents the number of genes, the larger the node, the higher the number of genes contained in it. Red‐coloured nodes symbolise genes stronger expressed in the *kdmA* mutant pointing to a direct or indirect negative regulatory role of the H3 demethylase for expression of these genes. In contrast, blue‐coloured nodes represent genes for which mRNA levels are higher in the wild type pointing to a positive regulatory role of KdmA in this case. Two main pathways were highly over‐represented in our GSEA, namely gene ontology (GO) terms relating to cellular energy production like genes involved in mitochondriondrial functions, the production of storage compounds such as trehalose or glycogen and genes required to counteract or to respond to the presence of hydrogen peroxide. The genes belonging to these cellular processes are represented as red‐coloured node‐centres in Fig. 6. Another large category of genes fall into GO terms associated with ribosome structural constituents, assembly of the components (nucleolus), and export from the nucleus as well as general protein translation functions. Related to this are the overexpressed genes involved in protein degradation (proteasome function and endopeptidases) and metabolism of branched‐chain amino acids. Higher mitochondrial activity, protein production and turn‐over may produce some internal redox imbalances resulting in upregulation of stress‐related genes. Thus, the latter function may be indirect and a consequence of higher respiratory stress. Overall, KdmA and possibly also its demethylation function is required to keep this subset of genes to a moderate or low level. In contrast, KdmA also seems to have a positive direct or indirect transcriptional role because in GSEA, we detected genes that were downregulated in the mutant (higher in the wild type, represented as blue‐coloured centres of the nodes in Fig. 6). Besides nucleosides metabolism and ethanol catabolism, these down‐regulated genes belong to the category of SM and penicillin biosynthesis, are significantly lower in the mutant under tested conditions. In our targeted gene analysis, we also found lower transcription of *ipnA*, one of the genes necessary for penicillin formation, in the mutant (compare Fig. 4). This lower expression in the mutant occurs despite higher H3K36me3 levels (compare Fig. 5) and thus points, in this specific case, to a negative role of H3K36me3 and a positive role for KdmA in *ipnA* early transcription.

**Figure 7:**
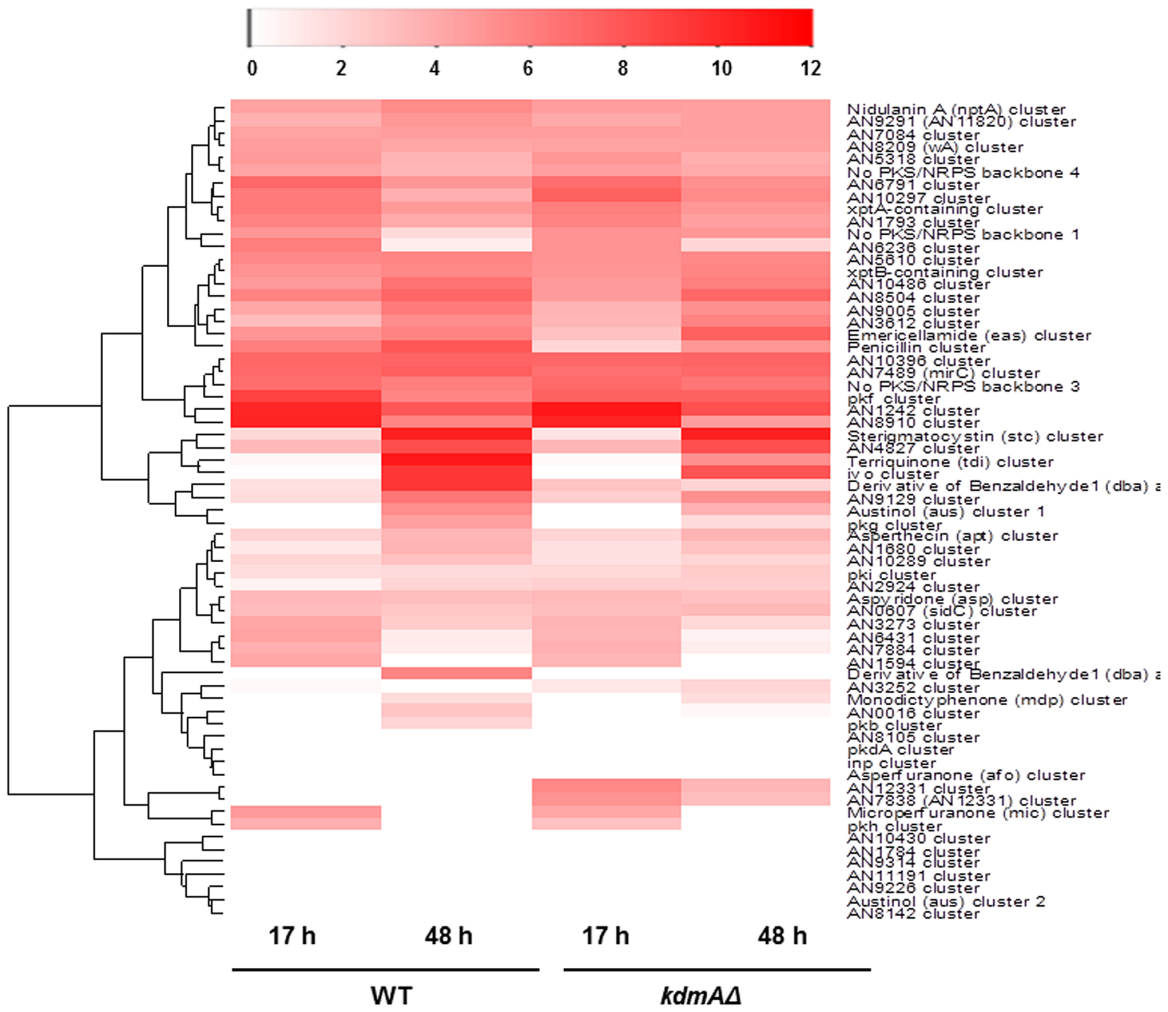
*K* *dm* *A* deletion alters expression pattern of SM cluster genes in secondary metabolism phase. Heat map for mean expression of previously annotated SM clusters (Inglis *et al*., 2013). Colour codes represent log_2_ of read counts per kb of exon per million total library reads (RPKM), the colour scales are located above the maps.

In order to better understand the role of KdmA in regulation of genes during SM, we extracted in GSEA genes differentially regulated in wild type and *kdmA*Δ during the SM phase (48 h, represented as node‐border colours in Fig. 6). The upregulation effect in the *kdmA* mutant of genes involved in the GO categories previously seen in PM (mitochondria, ribosomal functions, etc.) were largely maintained under these SM conditions (node‐borders coloured in red, KdmA function as repressor) but strikingly, GO terms associated with SM were downregulated in the mutant (enhanced in the wild type, blue node‐border colours). This is in agreement with our previous targeted analysis that also showed downregulation of induced *aflR and ipnA* levels in 48 h cultures (SM condtions, Fig. 4 and 6). So, although KdmA acts as repressor of some PM genes, it seems to be required for the full expression of SM genes. To verify this, we examined other predicted and annotated secondary metabolite gene clusters in more detail. A heat‐map of all predicted SM gene clusters (Inglis *et al*., 2013) is presented in Fig. 7. In this analysis, SM cluster mean expression was calculated based on RNAseq read counts normalised to the gene length and to the number of genes within the cluster (Inglis *et al*., 2013). We set the threshold for background expression to log_2_∼7 RNAseq reads corresponding to the number of reads obtained from the very low expressed cluster for asperthecin (APT) biosynthesis in the 48 h cultures (SM conditions). From the annotated 71 gene clusters associated with non‐essential secondary metabolic functions, 19 of these clusters were expressed significantly above the cut‐off value already under conditions of active growth, that is 17 h cultures corresponding to primary metabolic conditions (Fig. 7). This again undermines that ‘typical’ SM conditions do not exist and obviously different clusters need different growth or incubation conditions for expression of their cognate genes. In our case, we refer to SM conditions as those leading to high expression of the extensively studied model ST cluster. Only two out of the 19 SM clusters expressed already under PM conditions were KdmA dependent and repressed when the demethylase was missing. One of the repressed clusters carries penicillin biosynthesis genes, and this supports our control measurement by qPCR in which *ipnA* was also downregulated in the *kdmA*Δ mutant. The second cluster is coding for emericellamide (EAS) biosynthesis as described by Wang *et al*. (Chiang *et al*., 2008). In these two clusters, KdmA appears to act as transcriptional co‐activator under the conditions of active growth. Remarkably, the predicted cluster around AN12331, which is one of the roughly 40 orphan gene clusters without a known cognate metabolite and no expression under any of the classical culture conditions (neither PM nor SM conditions), is highly expressed in the *kdmA* mutant. Clearly, under these conditions, KdmA directly or indirectly reduces transcript abundance of gene in this gene cluster whereas it positively acts for PEN and EAS gene transcription. Interestingly, H3K36me3 levels were increased for *ipnA*, one tested gene belonging to the PEN cluster (Fig. 4), in the mutant under PM conditions. This shows that increased methylation in H3 at K36, generally considered a positively acting histone mark, is not necessarily associated with higher transcriptional activity of this gene.

Under our SM conditions, the effect of *kdmA* absence remains when we analyse the PEN cluster, that is also under these conditions KdmA function is required for full expression. Such a pattern can also be found at the terrequinone A (AN8513‐AN8520), the *dba*‐F9775 hybrid cluster (AN7896‐AN7916) and AN0016, *pkg*, *pkb* clusters with unknown biosynthetic function. However, also under SM conditions, we found opposing KdmA function, that is as transcriptional repressor in AN12331, AN9314 and the pkf gene clusters. So, under our conditions of SM, in six clusters KdmA has positive functions and in three clusters it displays negative functions. This is a clear indication that KdmA function influences transcription in positive and negative ways and this feature is locus and condition dependent.

### Manipulation of *kdm* *A* expression reveals genetic and environmental interactions including lethality under light

Deletion in a haploid *A. nidulans* strain shows *kdmA* is not essential; indeed tests across a range of carbon and nitrogen sources did not reveal any conspicuous growth or development phenotypes (Fig. S2). Most *A. nidulans* laboratory strains, including the transformation recipient strain used here, carry a partial loss of function mutation (defective nuclear import signal) in the developmental regulator *velvetA* (*veA*1) that arose through inadvertent selection (Käfer, 1965; Mooney and Yager, 1990; Mooney *et al*., 1990; Bayram *et al*., 2008b; Calvo, 2008). Although the wild‐type favours sexual development in the dark and requires light to promote asexual development (conidiation), *veA1* mutants show strong, uniform conidiation regardless of lighting due to the disrupted N‐terminal nuclear localisation sequence, with delayed and diminished sexual development (Champe *et al*., 1994; Kim *et al*., 2002). In order to assess any potential role for *kdmA* in sexual development, light response or SM, it was therefore necessary to generate a *kdmAΔ* strain in a *veA* ^+^ background.

**Figure 8:**
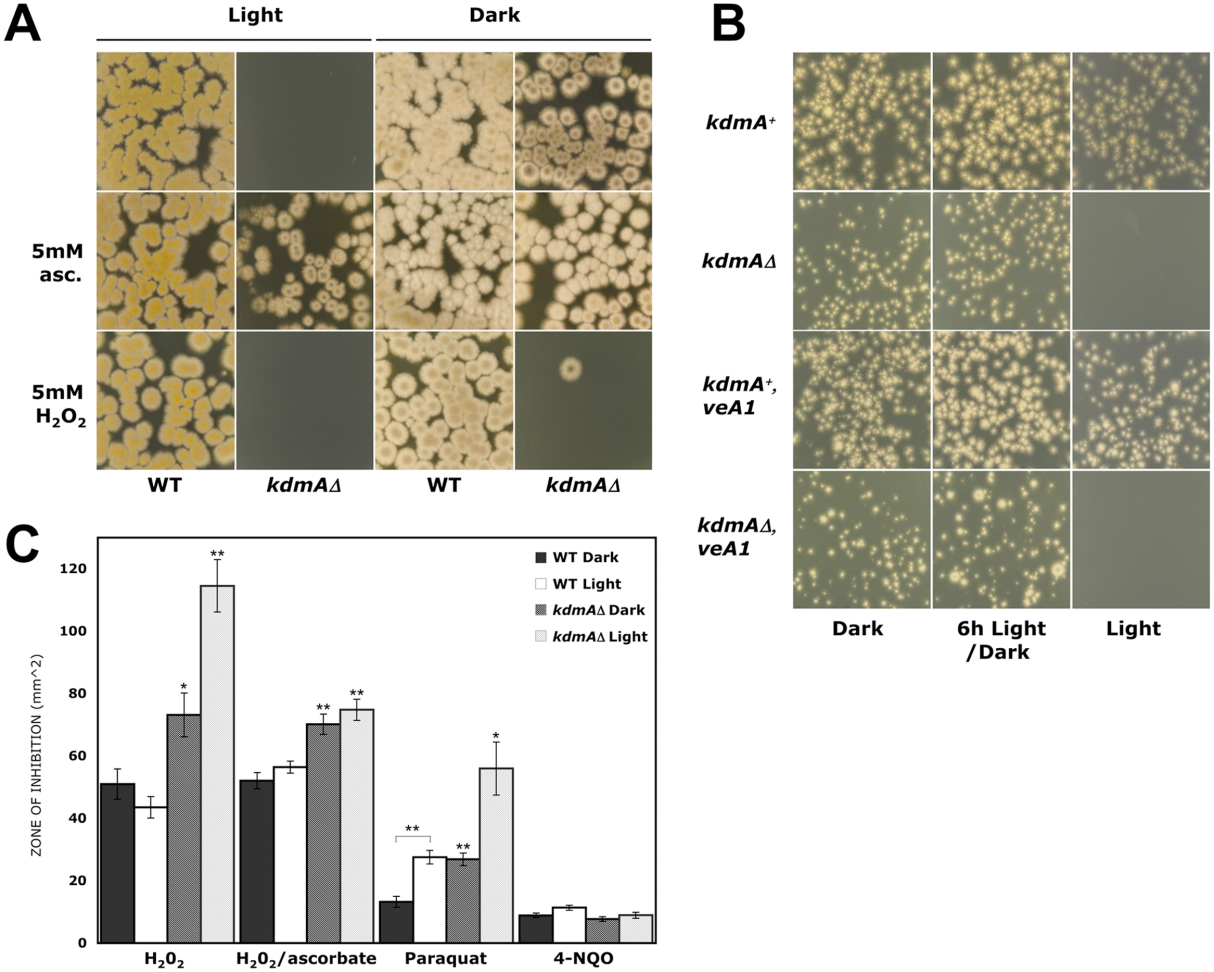
Deletion of *kdm* *A* causes light lethality and sensitivity to oxidative stress during vegetative growth. A. *kdmAΔ* (LN12211) and control WIM126 strains were inoculated at low density on complete medium alone, or with added ascorbate or hydrogen peroxide as indicated, and incubated in constant light or dark for 2 days at 37°C. B. *kdmAΔ* (LN12211, LN12063) and control strains (WIM126, MH11037) in *veA^+^* and *veA1* backgrounds were inoculated at low density (∼ 300 conidia per plate) and incubated either in constant dark or light for 24 h, or shifted from light to dark after 6 h. C. Zone of inhibition plate test for *kdmAΔ* (LN12211) and control (WIM126) strains (see *Experimental procedures*). The zone of inhibition area was measured and averaged for three wells across two plates for each treatment. H_2_0_2_/ascorbate is H_2_0_2_ added to plates containing 5 mM ascorbate. Errors are standard error of the mean. All statistical tests are comparisons between *kdmAΔ* and wild‐type in the same lighting condition and are significant by Student's *t*‐test at *P* < 0.05 (*) or *P* < 0.01 (**), except for wild‐type on paraquat, as indicated, which shows a significant response to light.

After outcrossing to a wild‐type strain and scoring progeny for segregating markers, including *pyrG^+^* as a proxy for the *kdmA* gene replacement, it was evident that no *kdmAΔ* and very few *veA1* progeny were present (*kdmA* is closely linked to *veA* on chromosome VIII), whereas all other tested genetic markers segregated independently in the expected Mendelian ratios. Extensive testing traced the basis of this bias to the presence of light. *kdmAΔ* ascospores or conidia plated at low density on complete medium (up to approximately 10 per cm^2^) under fluorescent white light failed to form colonies (Fig. 8A), whereas high‐density inoculation under light resulted in poor, irregular growth and impaired development. Incubation in the dark allowed recovery of all genotypic classes in the expected proportions, confirming the phenotype is a specific interaction between light and *kdmA* genotype.

Shifting plates from dark to light following inoculation showed that *kdmAΔ* strains were only susceptible after completion of germination. Conidia allowed to germinate under light for 6 h then shifted to dark for a further 18 h were indistinguishable from constant dark‐grown colonies, whereas light exposure after germination resulted in cumulative growth retardation (Fig. 8B). Sensitivity was not strongly wavelength dependent, as similar inhibition was seen under both blue and red light filters (Fig. S3).

*kdmA* expression was measured by quantitative reverse transcription polymerase chain reaction (RT‐PCR) in vegetative and developmental conditions, and in specific response to light exposure. mRNA increased slightly after acquisition of developmental competence and considerably during development (∼ 10‐fold during conidiation), but there was no evidence for light‐mediated induction of *kdmA* (Fig. S4).

A copy of *kdmA* under control of the *xylP* xylose inducible promoter was integrated at the *yA* locus. Crossing to a *kdmAΔ* strain and varying exposure of plated ascospores to light and xylose showed ectopic expression was able to fully restore light tolerance (Fig. S5). Overexpression in solid media resulted in secretion of an intense red/purple pigment into the medium beginning at around 36 h of growth (supplementary Fig. S6B). Pigment production was associated with derepressed sexual development, which is usually restricted to aerial growth, with numerous cleistothecia nurse (Hülle) cells produced beneath the agar surface (Fig. S6A). Dense profusions of pigment could be seen emerging from some of these Hülle cells at foci of maximal hyphal density (Fig. S6C). Targeted mass spectrometry of secondary metabolites using the multi metabolite method did not reveal any of the metabolites detected by this method.

### Lethality of *kdm* *A* *Δ* in light is due to chronic oxidative stress

As light is a well‐known pro‐oxidant (Gourmelon *et al*., 1994; Grzelak *et al*., 2001; Rossel *et al*., 2007; Ziegelhoffer and Donohue, 2009), antioxidants were tested for their ability to rescue the light lethality. Addition of ascorbate fully restored growth to *kdmAΔ* strains grown at low density under light (Fig. 8A). As would be expected if lethality were due to oxidative stress, loss of *kdmA* also resulted in greater sensitivity to oxidants such as H_2_O_2_, KO_2_ and paraquat in the dark, which was further exacerbated under light (Fig. 8C).

Light had no effect on wild‐type tolerance to H_2_O_2_ but clearly enhanced sensitivity to paraquat. Redox cycling by paraquat generates superoxide (O_2_●^‐^), which is subsequently converted to H_2_O_2_ and O_2_ either spontaneously or through the activity of superoxide dismutase (Halliwell, 2006). This suggested a synergistic action of light and superoxide is particularly damaging. Superoxide levels were examined by staining with dihydroethidium, either in constant dark, or dark followed by a 30 min treatment with light or exogenous superoxide in the form of aqueous KO_2_ (Fig. S7). Fluorescence was significantly higher for *kdmAΔ* under light relative to wild‐type, and to *kdmAΔ* in the dark (*P* < 0.05, Student's *t*‐test), consistent with an impairment in oxidative stress response. Although superoxide levels also increased in wild‐type in response to light exposure, only *kdmAΔ* was measurably affected by KO_2_ treatment. This suggests wild‐type is better able to cope with both a sustained (light) and acute (KO_2_) oxidative challenges.

Light could interact with a sensitised phenotype through photodynamic generation of reactive oxygen species (ROS). The thiazine dye Methylene Blue (MB) is widely used as a redox‐cycling indicator and photosensitiser (Oz *et al*., 2011) and has also been reported to have antioxidant activity (Zhang *et al*., 2006; Atamna *et al*., 2008). MB at 5 μM has been shown to effect photodynamic destruction of pyridoxine in *A. nidulans* growth medium, though without direct inhibition of a wild‐type strain (Osmani *et al*., 1999). We found that light‐dependent inhibition of wild‐type was evident at a concentration of 100 μM (Fig. S8). In contrast, 10 μM MB rescued the *kdmAΔ* light‐lethal phenotype, and no photodynamic action against the strain was apparent at higher doses. This suggests that, in contrast to wild‐type, MB acts predominantly as an antioxidant in the mutant and further supports the hypothesis that lethality is due to an oxidative imbalance.

To determine if light sensitivity is a common, undetected phenotype of stress response mutants, we generated and analysed a number of gene deletion strains for light sensitivity including the catalase genes *catA* and *catB*, mitochondrial SOD *sodM*, *fhdA*, *msnA*, flavohemoglobin *fhbA*, the alternative oxidase *aodA* and two‐component stress response regulator *srrA*. Of these, one mutant, *srrA,* showed a clear inhibition when incubated on complete medium under constant light, with mean colony area reduced to around 10% of dark incubated controls over the first 30 h of growth (data not shown).

### Transcriptional profiling of *kdm* *A* deletion under light

Whole genome microarray profiling was performed to address transcriptional responses to light on solid medium (in contrast to the conditions of submerged growth reported above). A clear response to light was detected in both wild‐type and *kdmAΔ*, despite a relatively low dynamic range of differential gene expression [63 genes were found to be regulated twofold or greater in *kdmAΔ* under light at *P* < 0.05 after correction for multiple testing, with a maximum absolute change of around ninefold (Fig. S9 and processed microarray data in Supplemental Table ST3)]. Most obvious by Gene Ontology enrichment was induction of genes annotated as responding to stressors such as heat ROS.

For both up‐ and downregulated wild‐type light responses, approximately 70% of genes were concordantly regulated in the *kdmAΔ* strain. Gene activity discordant between wild‐type and *kdmAΔ* or exaggerated in the mutant was examined in detail as this could underlie the observed light sensitivity. Few genes that were upregulated in wild‐type in response to light remained uninduced in *kdmAΔ* (Fig. S9). This class included metacaspase *casA*, MAP kinase kinase *mkkA* and the plasma membrane ATPase *pmaA*. Genes showing higher or unique differential expression in *kdmAΔ* under light were far more common and were significantly enriched for drug resistance factors (transporters *atrB*, *atrD, atrG* (AN0771), AN0732, AN2531, AN3952 and AN6581) and other stress response genes including five putative heat shock genes.

## Discussion

The *kdmA* gene encodes the sole member in *A. nidulans* of the highly conserved KDM4 family of JmjC domain lysine demethylases. Although the catalytic JmjC domain of *S. cerevisiae* Rph1 (regulator of photolyase 1) and *Aspergillus* KdmA are similar and contain functionally conserved residues, domain architectures differ in that Rph1/Gis1 have C_2_H_2_ DNA binding domains rather than the more general affinity AT‐hooks and PHD zinc finger of KdmA. In this regard, KdmA is similar to orthologues of higher eukaryotes that have only the PHD zinc finger (e.g. *Caenorhabditis elegans* JMJD2A) or PHD and Tudor (e.g. mouse KDM4A) interaction domains. However, the Tudor domain is not present in KdmA. Given the divergence in DNA binding domains and the fact that expression of *A. nidulans* photolyase *cryA* does not correlate closely with *kdmA* levels (data not shown), *A. nidulans* and *S. cerevisiae* downstream targets may not be conserved. Nevertheless, it is interesting that deletion of *cryA*, which acts as both photoreceptor and repair enzyme, also causes induction of Hülle cell development and secretion of a purple/pink pigment in liquid culture, similar to *kdmA* overexpression (Fig. S6C) (Bayram *et al*., 2008a). Further study is required to determine if *kdmA* overexpression phenotypes are mediated in part through *cryA* and to test for integration with known environmental sensing and developmental pathways.

All characterised members of the KDM4 family are capable of removing di‐ and trimethyl groups from lysines 9 and 36 of histone H3. H3K9me2 and me3 are modifications mostly associated with silent constitutive or facultative heterochromatic regions in which HP1 with its CD recognises these marks and thereby helps to form and maintain repressive structures (Bannister *et al*., 2001; Lachner *et al*., 2001). This classical view coming from studies in *Drosophila* and mammals has been revised as recent progress has shed light on additional roles in active transcription for the HP1 isoforms HP1b and HP1c where they colocalize with elongating PolII, chromatin chaperones and transcription factors (Li *et al*., 2002b; Greil *et al*., 2003; Grewal and Moazed, 2003; De Lucia *et al*., 2005; Kwon and Workman, 2011). Also for this positive function HP1 proteins still require H3K9 methylation, and it has been shown that H3K9me3 is enriched in some actively transcribed gene bodies (Turck *et al*., 2007; Liu *et al*., 2010; Roudier *et al*., 2011). Moreover, in *Drosophila*, HP1 directly interacts with and recruits the dKDM4a demethylase (a KdmA homologue) to a subset of heterochromatic genes thereby linking the H3K9me3 and H3K36me3 chromatin marks (Liang *et al*., 2011; Lin *et al*., 2012). Thus, KDM4 family demethylases serve at least two functions with opposing transcriptional readouts: in one case it interacts with HP1‐type proteins in silent heterochromatic regions, demethylates H3K9me3, and this results in loss of silencing and possibly gene activation. In the opposite pathway, it is also recruited to H3K9me3 by HP1, but it demethylates H3K36me3, a positively acting mark. This function would reinforce silencing by not touching the negatively acting (H3K9me3) but removing the positively acting (H3K36me3) mark. In our transcriptional analyses comparing wild type and *kdmA*Δ cells, we in fact see this bipartite pattern: genes that lose and genes that gain transcriptional activity when KdmA is absent. Interestingly, in submerged cultures, we see a clear separation of these activities between the two metabolic states tested in our studies. Under conditions of PM and active growth, we see higher activity of several classical housekeeping genes in the mutant (e.g. genes with predicted mitochondrial functions or involved in protein translation and stability). This result is consistent with the function of KdmA as transcriptional repressor. However, at the moment, we do not have data that would allow us to distinguish between repressor functions of KdmA by H3K36me3 demethylation or by binding to DNA as transcriptional repressor (or by indirect effects). ChIP data with increased H3K36me3 levels in the mutant at two loci belonging to secondary metabolite gene clusters (*aflR* and *ipnA*) strongly suggest that KdmA acts in fact directly as H3K36 demethylase. This function could be exclusively or in addition to a direct repression function of KdmA. Experiments are under way in which we are ChIPing wild‐type and enzymatically disabled tagged versions of KdmA to better understand the repressive function of this protein.

In this context, it is noteworthy that increased H3K36 methylation is not necessarily only associated with active transcription. Although we do see at several tested loci an increase in H3K36 methylation when the gene is induced, there is also a transcription‐independent increase of H3K36me3 seen for example at the *aflR* locus when KdmA is depleted. This is an important observation as it shows that H3K36me3 is not simply the consequence of transcriptional activity by PolII interaction as reported before (Smolle *et al*., 2013), but H3K36 methylation – and probably Set2 recruitment – occurs also independently of transcription. Although there is no transcriptional consequence of the increased H3K36 methylation levels in the SM gene clusters, we see higher transcript abundances when KdmA is deleted in many PM and housekeeping genes. Thus, KdmA in this case functions as negative transcriptional modulator maintaining a balanced network.

Where and to what extent KdmA functions as transcriptional co‐activator or facilitator, the mechanistic basis is even more obscure at the moment. This positively correlated function of KdmA is seen in genes belonging to secondary metabolite gene clusters that are activated in stationary cultures. We have previously shown for some selected SM gene clusters that HepA and the H3K9me3 methyltransferase ClrD (homologue of *N. crassa* Dim5) participate in silencing. The interaction between KDM4‐family members JHDM3A and HP1 is well established in *Drosophila* and mammals, and in these systems HP1 – via its H3K9me3‐binding modules – recruits KDM4A and stimulates the H3K36 demethylating activity of KDM4A (Lin *et al*., 2008; 2012). In this pathway, loss of HP1 results in increased H3K36me3 levels and higher transcript rates of target genes. Although this has not been tested in our work, we consider the KdmA–HepA interaction as a possible mechanism underlying the observed upregulation effect of several secondary metabolite gene clusters in HepA deletion strains.

However, this pathway does not function in both directions, as loss of KdmA does not lead to upregulation of SM genes but, conversely, to loss of transcription. Also on solid complex media expression from six of the 11 clustered genes responsible for biosynthesis of the secondary metabolite monodictyphenone (MDP) was significantly downregulated in the mutant. Interestingly, this was also the case for two xanthone prenyltransferases that are required for MDP biosynthesis but located outside of the gene cluster. MDP was recently identified as overproduced in a *cclA* (H3K4me2/3 methylation) mutant (Bok *et al*., 2009b; Chiang *et al*., 2010), associated with a reduction in both H3K9me3 and H3K4me2/3 at deregulated genes. This suggests that KdmA may be directly required for cluster expression through removal of repressive H3K9me2/3 marks, limiting heterochromatin protein 1 (HepA) occupancy. This positive function of KdmA may be similar to the antagonism found between HP1 and KDM4 (JHDM3A) at certain heterochromatic loci (Klose *et al*., 2006b). In this case, overexpression of the demethylase abrogates HP1 recruitment to heterochromatic sites and loss of the demethylase would consequently increase HP1 occupancy and repression. Thus, both mechanisms may be applicable to *A. nidulans*, but additional studies will be necessary to clarify if also in *A. nidulans* HepA and KdmA interact.

Unfortunately, due to contradictory results obtained with two different H3K9me3 antibodies regularly used in our laboratory (produced by Abcam and Active Motif), we were not able to unequivocally determine the degree of H3K9me3 in the studied SM cluster genes. Preliminary analysis of ChIP‐seq data (A. Gacek, H. Berger, Z. Lewis and J. Strauss, unpublished observations), which were obtained comparing the precipitation profiles of these two most frequently applied commercial H3K9me3 antibodies, suggest that in *A. nidulans*, the Abcam antibody to some extent also recognises H3K36me3. The alternative Active Motif antibody shows a distinct pattern of H3K9me3 at many different loci including some SM gene clusters under the applied conditions, and these data will be reported elsewhere with a detailed bioinformatic analysis.

Notwithstanding these uncertainties in the function of KdmA as H3K9me3 demethylase, we propose that its main role is to demethylate H3K36me3. This idea is based on our findings that this latter mark is by far the most common methylation mark on our identified bulk peptides. Together with H3K36me2 (which was detected by histone mass spectrometry but not tested here by ChIP), H3K36me3 is present on around 90% of identified peptides isolated from actively growing cultures. As mentioned above, this histone mark is traditionally associated with actively transcribed genes, and the dominance of these marks correlates well with a strong transcriptional activity under these growth conditions. In this context, it is not surprising that deletion of *kdmA* in *A. nidulans* produces both positive and negative changes in transcriptional readouts and that the number of affected genes is different under different conditions. It must be emphasised that the analysis performed in this work cannot distinguish between direct and indirect effects of KdmA function on gene expression. To be able to differentiate between direct target genes of KdmA and indirect transcriptional effects, one would need genome‐wide KdmA localisation studies (ChIP‐seq) using a tagged functional version of the protein. In addition, on directly targeted genes, the protein may act as histone H3K36 demethylase and/or as transcriptional adaptor protein independently of its demethylase function and thus positively or negatively affecting transcription. To distinguish between these different potential functions of KdmA, the ChIP‐seq analysis would require tagged versions of different KdmA variants carrying mutations in the proposed functional domains (see Fig. 1). Finally, we cannot exclude that KdmA may act also as lysine‐demethylase on non‐histone substrates, as documented in other systems (Ponnaluri *et al*., 2009).

Despite the generally subtle growth and developmental consequences seen upon manipulation of *kdmA* expression under standard laboratory conditions, variation of the environment revealed dramatic phenotypes. Deletion resulted in lethality for isolated conidia incubated under fluorescent white light, generally considered a benign environmental cue that directs *A. nidulans* towards asexual conidiation and away from sexual development. Transcriptional profiling revealed that light provoked a broad stress response even in wild‐type, also evident in a recent study measuring the response of wild‐type to a shorter (30 min) light pulse (Ruger‐Herreros *et al*., 2011). Higher expression in the mutant of genes related to mitochondrial function would be consistent with unbalanced intracellular redox status thus leading to higher susceptibility against pro‐oxidants found in our phenotyping analysis of the mutant. Although experimental conditions were very different, data from the two studies were significantly correlated (Pearson's *r* = 0.54), suggesting a large proportion of light regulated expression is stable over the 0.5–2 h window.

It is clear that light can be both a developmental cue and a significant stress to *A. nidulans*. If *kdmA*Δ is lethal under light due to chronic oxidative stress, then it might be expected that induction of stress response genes is impaired in the mutant. This has been observed for deletion of other chromatin modification genes in *C. elegans* and *D. melanogaster* (Kirienko and Fay, 2010) and, in *A. nidulans*, for arginine methyltransferases *rmtA* and *rmtC* (Bauer *et al*., 2010) and histone deacetylase *hdaA* (Tribus *et al*., 2005). Transcriptional profiling of the *kdmA* deletion on solid medium identified a diverse group of genes implicated in stress response that are misregulated under light in the mutant. These ranged from signal transduction and ubiquitin proteasome components to factors directly involved in neutralisation of reactive redox species, such as peroxiredoxin and glutathione transferases. However, transcriptional misregulation of these candidates was, individually, modest in scale and the mutant phenotype may be due to additive effects, or post‐transcriptional regulation. We also have to consider that some differentially regulated genes in the *kdmA* mutant may simply respond to the increased oxidative stress of mutant cells under normal growth conditions and thus are not under direct KdmA control. Given the complex genotype‐by‐environment interactions seen between *kdmA* and SM production, and exogenously supplied modulators of oxidative stress, another possibility is that certain SMs are photodynamic directly contribute to light sensitivity in *kdmAΔ*.

This is not mutually exclusive with a generally impaired stress response and distinguishing between these hypotheses will require additional phenotypic and functional data.

**Figure 9:**
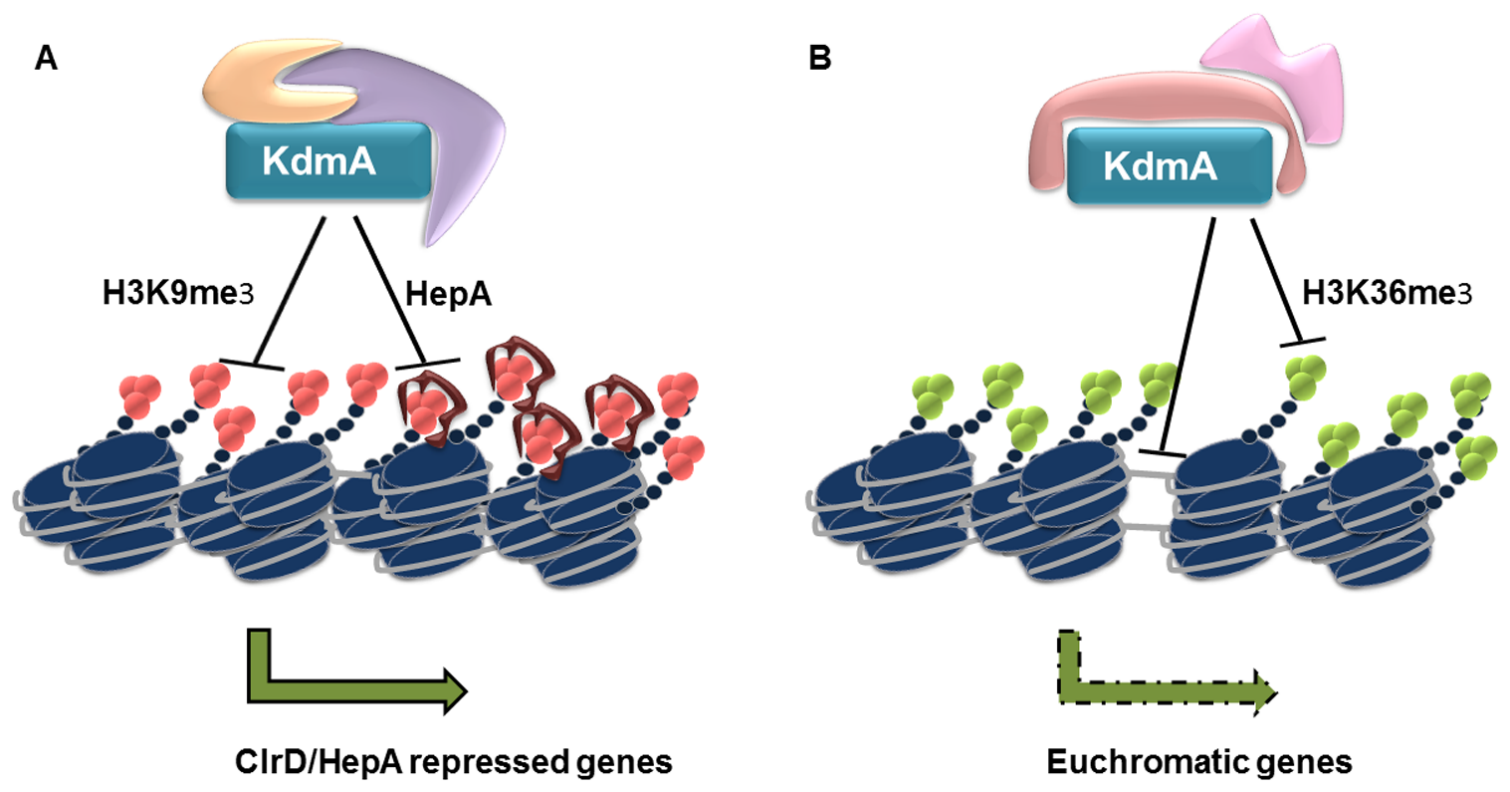
Possible model for the bipartite function of *K* *dm* *A*. A. In this scenario (e.g. SM gene clusters), KdmA would function as activator by removing trimethyl groups from H3K9 or by directly counteracting HepA binding. Enzymatic function and direct competition with HepA or other chromatin modifiers are not exclusive events. B. When KdmA acts as repressor of transcription its demethylases activity would lead to removal of H3K36me3 and subsequently, cryptic promoters within gene bodies would become active and block transcriptional elongation. Alternatively or additionally, KdmA, via its DNA binding domain, may directly repress transcription. Both mechanisms would lead to downregulation of transcriptional activity of the target genes.

Thus, *kdmA* plays a vital role in facilitating adaptation to ecologically relevant, stressful and competitive conditions encountered in its natural heterogeneous environment. Our data indicate that, on the molecular level, KdmA must have at least two functions: as an enzymatically active H3K36me3 demethylase as well as a transcriptional adaptor possibly mediating both transcriptional induction and repression at different sets of genes. In Fig. 9, we summarise our findings in a model and speculate that KdmA may act as antagonist of the silencing machinery in genes or gene clusters that are under the control of heterochromatic marks (HepA, ClrD). Furthermore, KdmA may act as transcriptional adaptor in addition to its function as demethylase or heterochromatic antagonist even at the same genomic location. To decipher these distinct functions, it will be necessary to study different KdmA mutants with defects in each of the predicted functional domains. Ongoing genome‐wide localisation studies with an active and enzymatically disabled version of KdmA will at least partially reveal already which of the two functions is exerted by KdmA at which locus, thereby improving our understanding of the role histone modifiers play in fungal metabolic und developmental regulation.

## Experimental procedures

### Strains, media, growth conditions and transformations

*Aspergillus nidulans* strains used in this study are listed in Supplementary Table 1. Experimental strains were obtained by sexual crosses or by transformation into TN02A25 (MH11037) *nkuAΔ* strain, which reduces the frequency of non‐homologous integration (Nayak *et al*., 2006), but then crossed to WIM126 to get veA+ or remove background markers. Genetic analysis was carried out using techniques as described by Todd *et al*. (2007b). DNA transformation of *A. nidulans* was performed according to Tilburn *et al*. (1983) and Andrianopoulos and Hynes (1988). KdmA deletion cassettes were constructed using DJ PCR or split‐marker fusion PCR (Kuwayama *et al*., 2002; Yu *et al*., 2004), with the *N. crassa* and *A. fumigatus* pyr‐4 genes as selectable markers, pyrG+ transformants were recovered after transformation into an *nkuA* strains (Nayak *et al*., 2006). Southern analysis confirming the deletion of *kdmA* is shown in Figure S11. AG004 *kdmAΔ* and WT *paba A1* were used unless stated otherwise. Other deletion strains screened for light sensitivity were made using deletion cassettes obtained from the Fungal Genetics Stock Center (McCluskey *et al*., 2010).

ANM minimal media, complete medium, supplements and growth conditions were as described by Todd *et al*. (2007a). For SM cluster gene expression, ChIP and HPLC‐MS/MS analysis 10 mM sodium nitrate otherwise ammonium tartrate at 10 mM was added as nitrogen source. YEG (0.5% yeast extract, 2% carbon source) medium was also used were indicated. For induction of the *xylP* promoter, xylose or xylan was used at the indicated concentrations.

For SM cluster gene expression, ChIP and HPLC‐MS/MS analysis spores in concentration 4*10^6^/ml were inoculated into 200 ml AMM and incubated at 180 r.p.m. for 17 h and 48 h.

Incubation was at 37°C except where noted otherwise. For ‘dark’ treatment, plates were loosely wrapped in aluminium foil within an unlit incubator. For ‘light’ treatment, plates were incubated at a distance of approximately 1 m from polycarbonate shielded F36W/840 triphosphor fluorescent white lighting (Sylvania Lamps).

### Phylogenetic analysis

JmjC domain proteins were sourced from the SMART database (Schultz *et al*., 1998; Letunic *et al*., 2009), aligned by Clustal multiple sequence alignment in MEGA version 5 (Tamura *et al*., 2011), analysed by Bayesian inference of phylogeny in MrBayes 3.2.1 (two independent runs of 1 million generations with a mixed amino acid model and a mixture of variant and invariant sites approximated by a four‐category gamma distribution) (Huelsenbeck and Ronquist, 2001; Ronquist and Huelsenbeck, 2003) and imported into FigTree (http://tree.bio.ed.ac.uk/software/figtree/) for graphical manipulation.

### Deletion and overexpression of *kdm* *A*

Oligonucleotides used are shown in Supplementary Table S2. The *kdmA* deletion construct was generated using two‐step fusion PCR to link *kdmA* flanking regions (primers *kdmA* S1 and S2, S7 and S8) to overlapping fragments of the *N. crassa pyr‐4* or *A. fumigatus pyrG* gene (primers Nc pyr‐4 S3 and S4, S5 and S6), and transformed as two linear split‐marker fragments (Catlett *et al*., 2003) or as linear deletion cassette (Yu *et al*., 2004). For *kdmA* overexpression, coding region was amplified by PCR (primers kdmA F4 OE and kdmA S1) and cloned into the HindIII and SmaI sites of the xPyA‐3 vector (pCW7468) (Wong *et al*., 2009) to form plasmid pLN7807.

### Nucleic acid isolation

*Aspergillus nidulans* genomic DNA was extracted from frozen mycelia as described by Lee and Taylor (1990). For small‐scale preparations, a dilute spore solution was lysed by brief sonication (Branson Ultrasonics) and purified by phenol/chloroform extraction. Total RNA was isolated from filtered, flash frozen mycelia using a Fastprep bead‐beater (MP Biomedicals) and Trizol reagent (Invitrogen) following the manufacturers' protocol.

### Real‐time RT‐PCR

Total RNA was treated with DNAse I (Promega) and reverse transcribed into cDNA using the Promega Reverse Transcription System with random primers. PCR was carried out in a Corbett RG‐3000 thermocycler using Quantace SYBR Green SensiMix. Two to three biological replicates with three technical replicates were carried out, and differential expression was calculated using the comparative Ct method against a beta‐tubulin standard (*benA*).

### ChIP coupled quantitative PCR

Chromatin immunoprecipitation was modified from Bernreiter *et al*. (2007). Mycelia after 17 h and 48 h culture were cross‐linked for 15 min with 1% formaldehyde at room temperature, followed by 5 min quenching with 125 mM glycine. Chromatin was incubated with antibodies specific to H3K36me3 (Abcam, 9050), or Histone H3 C‐terminus (Abcam, 1791) and Dynabeads Protein A (Invitrogen). Precipitated DNA from two biological and two technical replicates was quantified by real‐time PCR according to protocol (Bio‐Rad) using iQ SYBR Green Supermix and normalized to input DNA. Primers used in quantitative PCR were HPLC purified and are shown in Supplementary Table 2.

### High throughput RNA sequencing (RNAseq) and analysis

Illumina sequencing libraries were made from RNA samples according to TruSeq RNA Sample prep kit v2 (Illumina) following the manufacturers protocol with 1 μg total RNA input. The 50 bp single end sequencing was performed using a HiSeq Illumina sequencer. Obtained sequences were de‐multiplexed, quality controlled and mapped on the *A. nidulans* genome assembly (A_nidulans_FGSC_A4_version_s10‐m03‐r07). Mapping was performed using Novoalign (NovoCraft), and reverse transcripts were counted using python script HTSeq (Anders *et al*., 2014). Normalisation and statistics were done using R/Bioconductor and the limma and edgeR packages, using mean‐variance weighting (voom) and TMM normalisation (Gentleman *et al*., 2004). A significance cut‐off of *P* < 0.05 (adjusted for multiple testing by the false discovery rate method) was applied for analysis.

Gene functional analysis used GSEA with 1000 gene set permutations (Subramanian *et al*., 2005), the Enrichment Map plugin for Cytoscape 2.8 (http://www.cytoscape.org/index.html) with minimum *P*‐value of 0.005 and false discovery rate Q‐value 0.1 (Merico *et al*., 2010; Smoot *et al*., 2011; Saito *et al*., 2012). R plots used the ggplot2 package (Wickham, 2009). Heatmaps of cluster transcription were calculated from the mean of normalised (sequencing libraries and exon length). Transcription levels are log2 read counts per kilobase of exon per million library reads (RPKM). SM clusters are annotated as described by Inglis *et al*. (2013).

### Secondary metabolites HPLC‐MS/MS analysis

The analysis of sterigmatocystin was performed using an HPLC‐MS/MS method for the determination of 186 fungal and bacterial metabolites described by Vishwanath *et al*. (2009). Detection and quantification in the Selected Reaction Monitoring (SRM) mode was performed with a QTrap 4000 LC‐MS/MS System (Applied Biosystems, Foster City, CA, USA) equipped with a TurboIonSpray electrospray ionization (ESI) (Applied Biosystems, Foster City, CA, USA) source and an 1100 Series HPLC System (Agilent, Waldbronn, Germany). Chromatographic separation was performed at 25°C on a Gemini C18 column, 150  ×  4.6‐mm i.d., 5 μm particle size, equipped with a C_18_ 4 × 3 mm‐i.d. security guard cartridge (Phenomenex, Torrance, CA, USA). Injection volume was 5 μl. For quantification, external calibration was performed using multi‐analyte standards prepared and diluted in a 1:1 mixture of extraction and dilution solvent.

All chemicals (LC gradient grade) were from J.T. Baker (Deventer, The Netherlands); ammonium acetate (MS grade) and glacial acetic acid (p.a.) were obtained from Sigma‐Aldrich (Vienna, Austria). Water was purified successively by reverse osmosis and a Milli‐Q plus system from Millipore (Molsheim, France). A certified standard for sterigmatocystin in acetonitrile was from Biopure Referenzsubstanzen GmbH (Tulln, Austria) and Sigma (Vienna, Austria).

### Analysis of HPTM by Western blot and LC‐MS/MS

Mycelia from o/n liquid submerged cultures were harvested by filtration and frozen in liquid nitrogen. Histones were acid extracted as previously described (Honda and Selker, 2008), suspended in Laemmli's SDS sample buffer and quantified with Pierce BCA Protein Assay (Thermo). 15 µg of purified histones, 1 μg of recombinant *Xenopus laevis* H3 as a negative control (Milipore, 14‐ 441) and 2 μg calf thymus histones (Sigma, H9250) as a positive control were separated on 15% SDS‐PAGE gel and subsequently transferred to nitrocellulose membrane (GE Healthcare) by electroblotting. Relevant histone modifications were detected with primary antibodies specific to H3K9me3 (Active Motif, 39161), H3K9me2 (Abcam, 1220), H3K27me3 (Abcam, 6002), H3K36me3 (Abcam, 9050), histone H3 C‐terminus (Abcam, 1791) and anti‐rabbit (Sigma, A0545) and anti‐mouse (Sigma, A9044) HRP conjugated secondary antibodies. Chemiluminescence was detected with Clarity^TM^ ECL Western Substrate and ChemiDoc™ XRS (Bio‐Rad).

For MS analysis, relevant histone H3 protein bands were cut out and digested in gel. The proteins were S‐alkylated with iodoacetamide and digested with ArgC (Roche). The peptide mixture was analysed using a Dionex Ultimate 3000 system directly linked to a Q‐TOF MS (Bruker maXis 4G ETD) equipped with the standard ESI source in the positive ion, DDA mode (= switching to MSMS mode for eluting peaks). MS‐scans were recorded (range: 150–2200 Da), and the six highest peaks were selected for fragmentation. Instrument calibration was performed using ESI calibration mixture (Agilent). For separation of the peptides, a Thermo BioBasic C18 separation column (5 μm particle size, 150*0.360 mm) was used. A gradient from 95% solvent A and 5% solvent B (Solvent A: 0.1% FA in water, 0.1% FA in ACCN) to 32% B in 45 min was applied, followed by a 15 min gradient from 32% B to 75% B that facilitates elution of large peptides, at a flow rate of 6 μl/min.

## Supporting information

Supporting information
