# Supplementary material for: KdmA, a histone H3 demethylase with bipartite function, differentially regulates primary and secondary metabolism in A spergillus nidulans
Source: Mol Microbiol. 2015 Apr 11;96(4):839–60. doi: 10.1111/mmi.12977 (PMC4949671; doi:10.1111/mmi.12977)
Supplement: Supplementary file 1 — Supporting information [file MMI-96-839-s001.zip › MMI_12977_supp-0001-SI.pdf]

Material and Methods Supplementary Information

Oxidative stress plate tests

Zone of inhibition tests were carried out after Maerker et al. (Maerker et al., 2005) with the following modifications. Supplemented 1% agar ANM cooled to 40°C was inoculated with 105 conidia ml-1 and plates poured immediately. After 6 hours growth at 37°C, agar plugs were excised to create wells, testing agents applied in 150 µl water and plates returned to 37°C for a further 18 hours in dark or light conditions as indicated. Plates were digitally scanned and the zone of inhibition measured in ImageJ (http://rsb.info.nih.gov/ij/) and averaged for three wells across two plates for each treatment.

Microarrays

Mycelium for kdmA overexpression or liquid culture kdmAΔ microarray experiments was from liquid cultures, inoculated to a density of 106 conidia ml-1 in 200 ml of supplemented ANM minimal medium in 1 L flasks. Cultures were grown for 16 h at 37°C shaken at 120 rpm then harvested by filtration. For overexpression, mycelia was washed and transferred to fresh media containing either 1% glucose or xylose as sole carbon source for a further 4 hours. For solid culture light exposure experiments, complete medium agar plates were spread with approximately 500 conidia and incubated at 37°C in the dark for 46 hours, then exposed to light or continued dark for a further two hours. Plates were immediately snap frozen in liquid nitrogen and mycelium was harvested by scalpel for RNA extraction.

RNA was reverse transcribed using the Invitrogen Superscript Indirect Labelling Kit with oligo d(T) primers according to the manufacturer's protocol. cDNA was purified using Zymo DNA CLEAN & Concentrator columns and labelled with Biosearch Qasar fluorescent dyes. Samples were competitively hybridised to version 2 PFGRC whole genome glass slide microarrays according to TIGR protocol M008. For all experiments two biological replicates as dye-swaps were hybridised for every sample (with the exception of a single comparison for dark-grown kdmAΔ).

Microarrays were scanned on an Axon GenePix 4100, the PFGRC supplied GAL file was aligned and manually checked and data was acquired using morphological opening correction. Raw data were imported into R (http://www.r-project.org/) and analysed using the limma package with normexp background correction (offset = 50) and duplicateCorrelation for duplicate probe spots, normalised to a wild-type reference sample (Smyth et al., 2005, Smyth, 2005, Ritchie et al., 2007, Smyth & Speed, 2003). An empirical Bayes moderated t-statistic p-value < 0.05 (adjusted for multiple testing by the Benjamini and Hochberg method) was used to assess differential expression by genotype or treatment. Further analysis was carried out in R, the TM4 MultiExperiment Viewer (Saeed et al., 2006, Saeed et al., 2003), and functional analysis used FungiFun (Priebe et al., 2011), the AspGD GO Term Finder (Arnaud et al., 2010) and REVIGO (Supek et al., 2011). Data are available from NCBI GEO under accession GSE38694.

Microscopy

For analysis of superoxide levels, conidia were inoculated at low density (103 ml-1) onto glass slides coated with 1% agar complete medium and grown at 25°C for 16 hours. DHE (Sigma) at 20 µm in 0.1% Tween-80 was overlaid and loaded for 30 minutes at 37°C. Treatments were applied for a

further 30 minutes and slides were immediately imaged on an Olympus FluoView FV1000 confocal microscope sequentially capturing transmitted light, UV and 2-hydroxyethidium at excitation 490 nm/emission 595 nm. Fluorescence was quantified in ImageJ for at least 20 images from three independent experiments.

For analysis of nuclear morphology, conidia were inoculated at low density (103 ml-1) onto glass slides coated with 1% agar complete medium and grown at 25°C for 12 hours, followed by 45 minutes of continued dark or light exposure at 37°C. Hyphae were fixed in 4% paraformaldehyde/PME and imaged by laser scanning confocal microscopy on an Olympus FluoView FV1000 with Z-dimension capture every 0.5 µm. Image stacks were analysed in ImageJ, measuring quantitative H1-GFP signal characteristics (e.g. mean, median and standard deviation of grey values) in a 3 µm2 region centred on each nucleus, or were converted to binary using a fixed threshold value to measure area and shape descriptors (e.g. circularity (4π(area/perimeter2)). Stated values therefore represent averages for multiple slices through each nucleus. A minimum of 28 nuclei (mean of 35) across two experiments was measured for each treatment.

Arnaud, M.B., M.C. Chibucos, M.C. Costanzo, J. Crabtree, D.O. Inglis, A. Lotia, J. Orvis, P. Shah, M.S. Skrzypek, G. Binkley, S.R. Miyasato, J.R. Wortman & G. Sherlock, (2010) The Aspergillus Genome Database, a curated comparative genomics resource for gene, protein and sequence information for the Aspergillus research community. Nucleic Acids Res 38: D420-427.

Maerker, C., M. Rohde, A.A. Brakhage & M. Brock, (2005) Methylcitrate synthase from Aspergillus fumigatus. Propionyl-CoA affects polyketide synthesis, growth and morphology of conidia. FEBS Journal 272: 3615-3630.

Priebe, S., J. Linde, D. Albrecht, R. Guthke & A.A. Brakhage, (2011) FungiFun: a web-based application for functional categorization of fungal genes and proteins. Fungal Genet Biol 48: 353-358.

Ritchie, M.E., J. Silver, A. Oshlack, M. Holmes, D. Diyagama, A. Holloway & G.K. Smyth, (2007) A comparison of background correction methods for two-colour microarrays. Bioinformatics 23: 2700-2707.

Saeed, A.I., N.K. Bhagabati, J.C. Braisted, W. Liang, V. Sharov, E.A. Howe, J. Li, M. Thiagarajan, J.A. White & J. Quackenbush, (2006) TM4 microarray software suite. Methods Enzymol 411: 134-193.

Saeed, A.I., V. Sharov, J. White, J. Li, W. Liang, N. Bhagabati, J. Braisted, M. Klapa, T. Currier, M. Thiagarajan, A. Sturn, M. Snuffin, A. Rezantsev, D. Popov, A. Ryltsov, E. Kostukovich, I. Borisovsky, Z. Liu, A. Vinsavich, V. Trush & J. Quackenbush, (2003) TM4: a free, open-source system for microarray data management and analysis. Biotechniques 34: 374-378.

Smyth, G.K., (2005) Limma: linear models for microarray data. Springer, New York.

Smyth, G.K., J. Michaud & H.S. Scott, (2005) Use of within-array replicate spots for assessing differential expression in microarray experiments. Bioinformatics 21: 2067-2075.

Smyth, G.K. & T. Speed, (2003) Normalization of cDNA microarray data. Methods 31: 265-273.

Supek, F., M. Bosnjak, N. Skunca & T. Smuc, (2011) REVIGO summarizes and visualizes long lists of gene ontology terms. PLoS One 6: e21800.

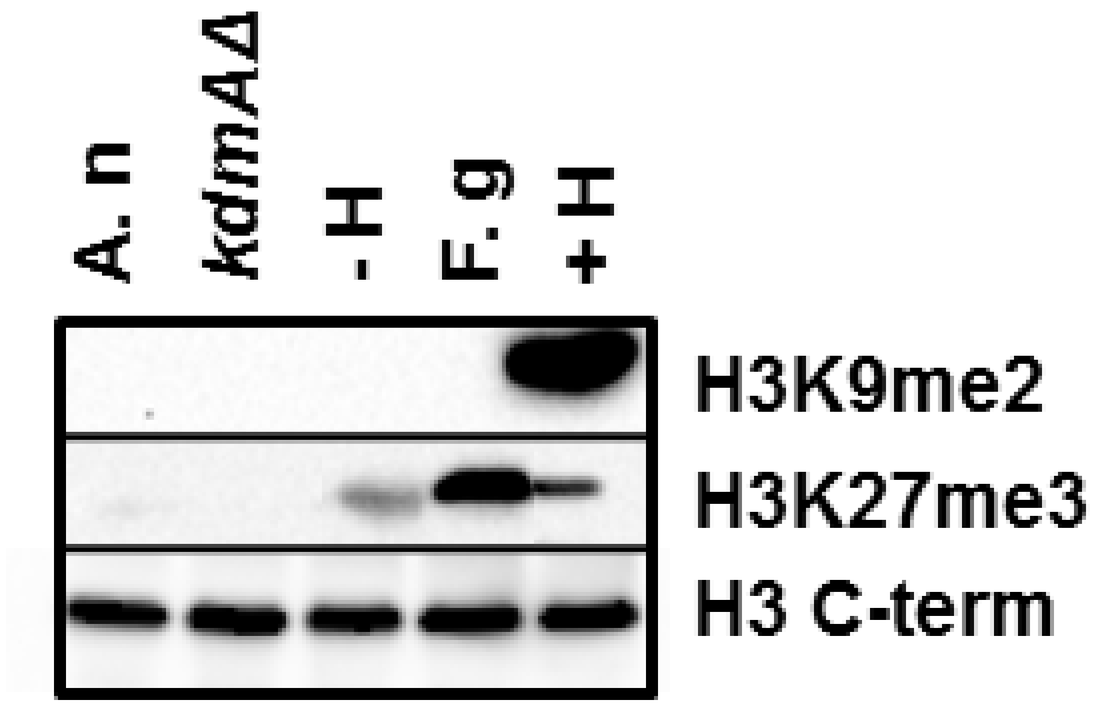

Supplementary Figure 1. Western blot with antibody specific to H3K9me2, H3K27me3 and histone H3- C term. 15µg of acidic extracted *Aspergillus nidulans* (A.n) and *Fusarium graminearum* (F.g) histones, 2 µg calf thymus histones (+H) and 1 µg of recombinant *Xenopus laevis* histone H3 (-H) were loaded.

A

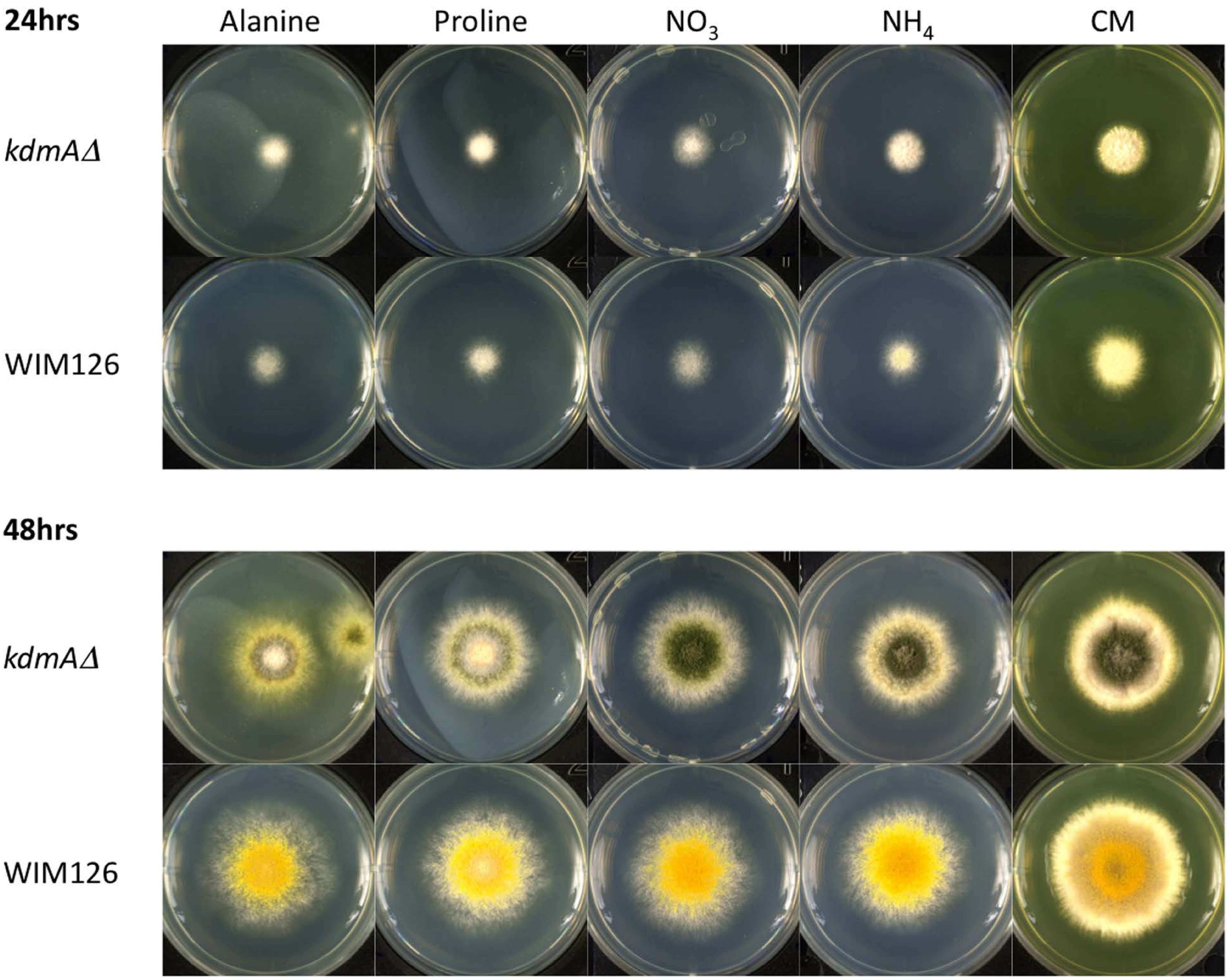

Supplementary Figure 2. Carbon and nitrogen utilisation tests for WIM126 and LN12211. Nitrogen (**A**, **B**; alanine, proline NO3, NH4) sources are 10 mM with 1% w/v glucose as carbon. Carbon sources (**C**, **D**; lactose, butyrate, galactose, glucose) are 1% w/v with 10 mM (NH4)2T as nitrogen in minimal medium base. CM is 0.5% yeast extract 2% glucose medium.

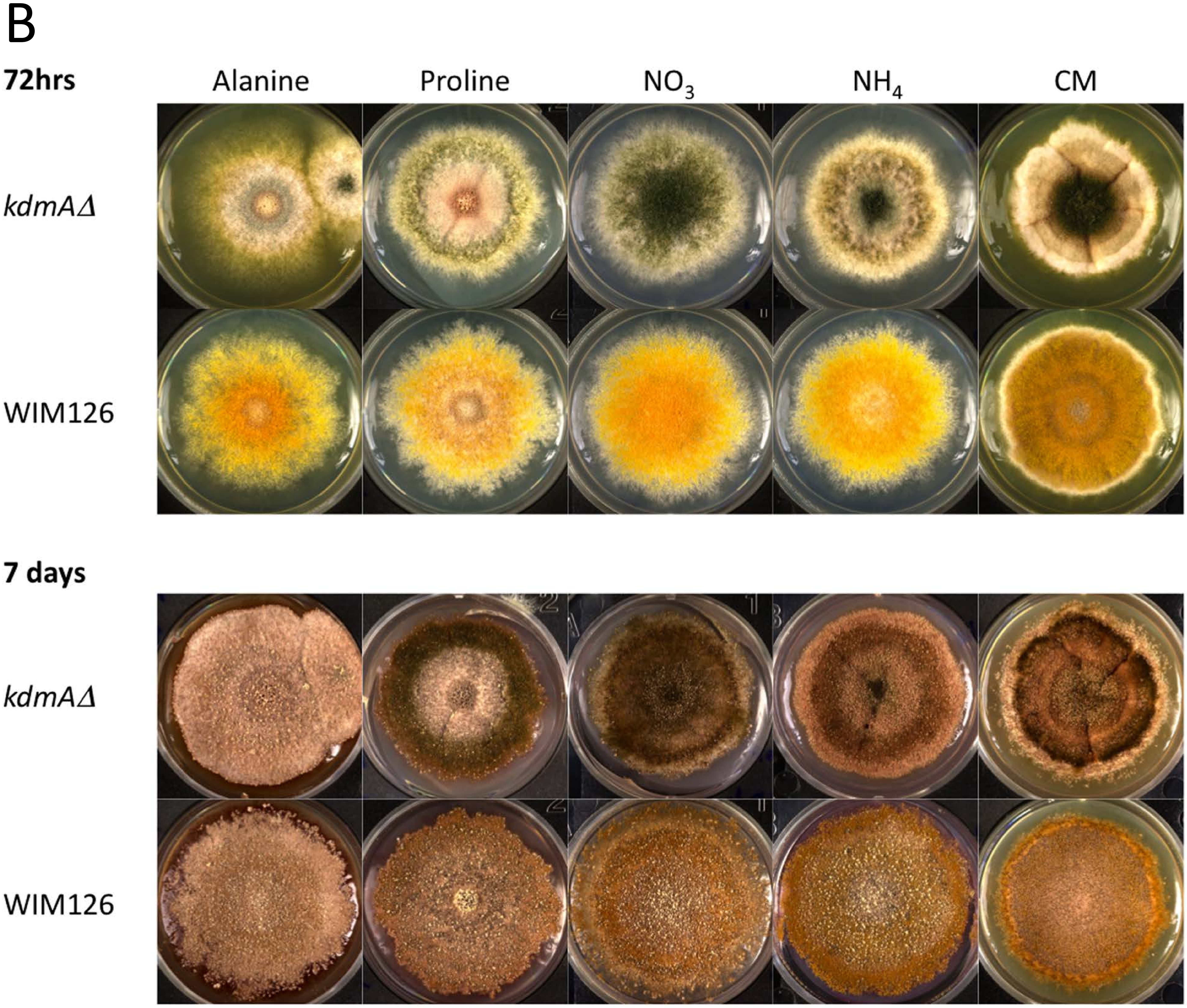
B
72hrs
Alanine
Proline
NO_3
NH_4
CM
kdmAΔ
WIM126
7 days
kdmAΔ
WIM126

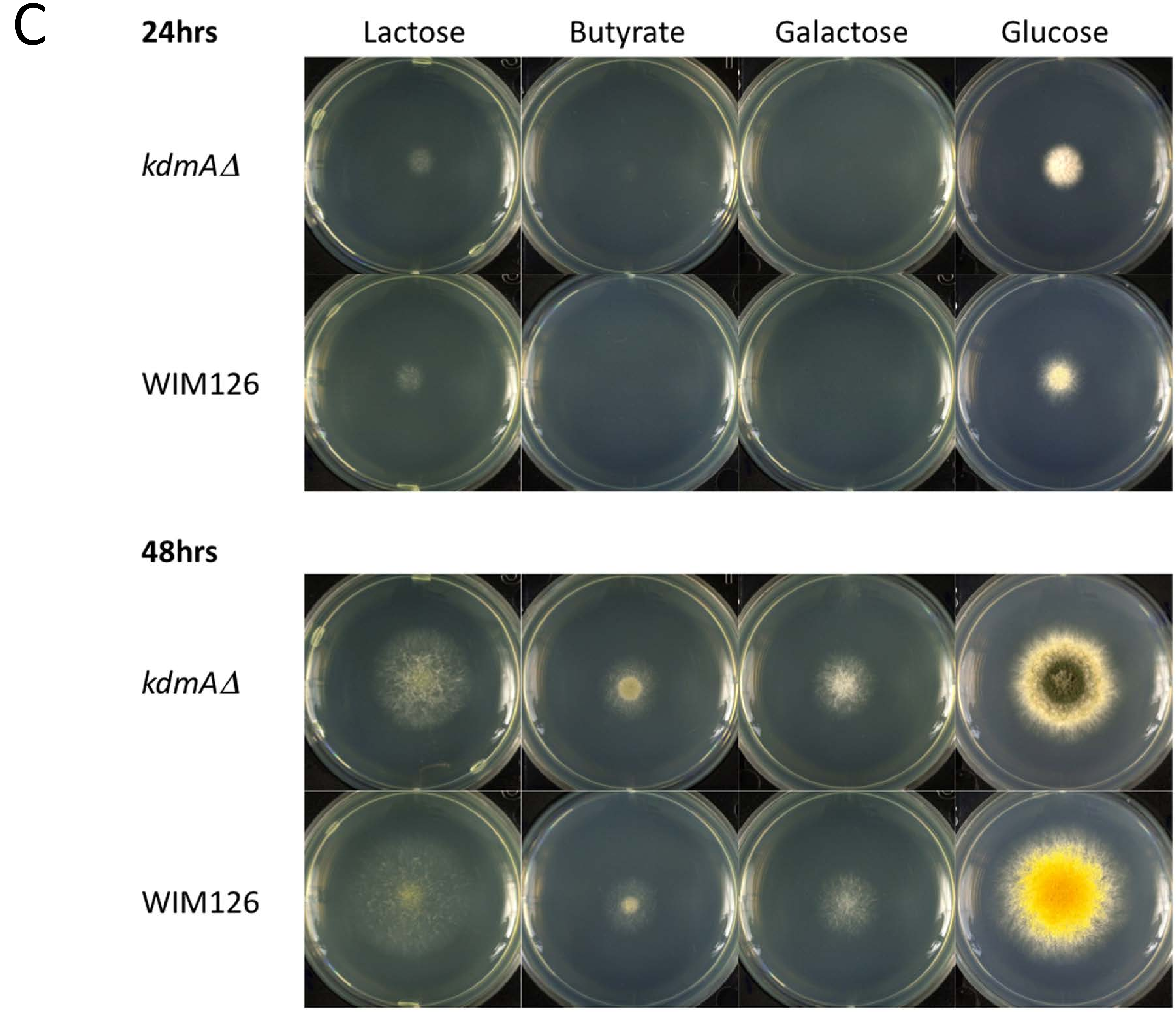
C
24hrs
Lactose
Butyrate
Galactose
Glucose
kdmAΔ
WIM126
48hrs
kdmAΔ
WIM126

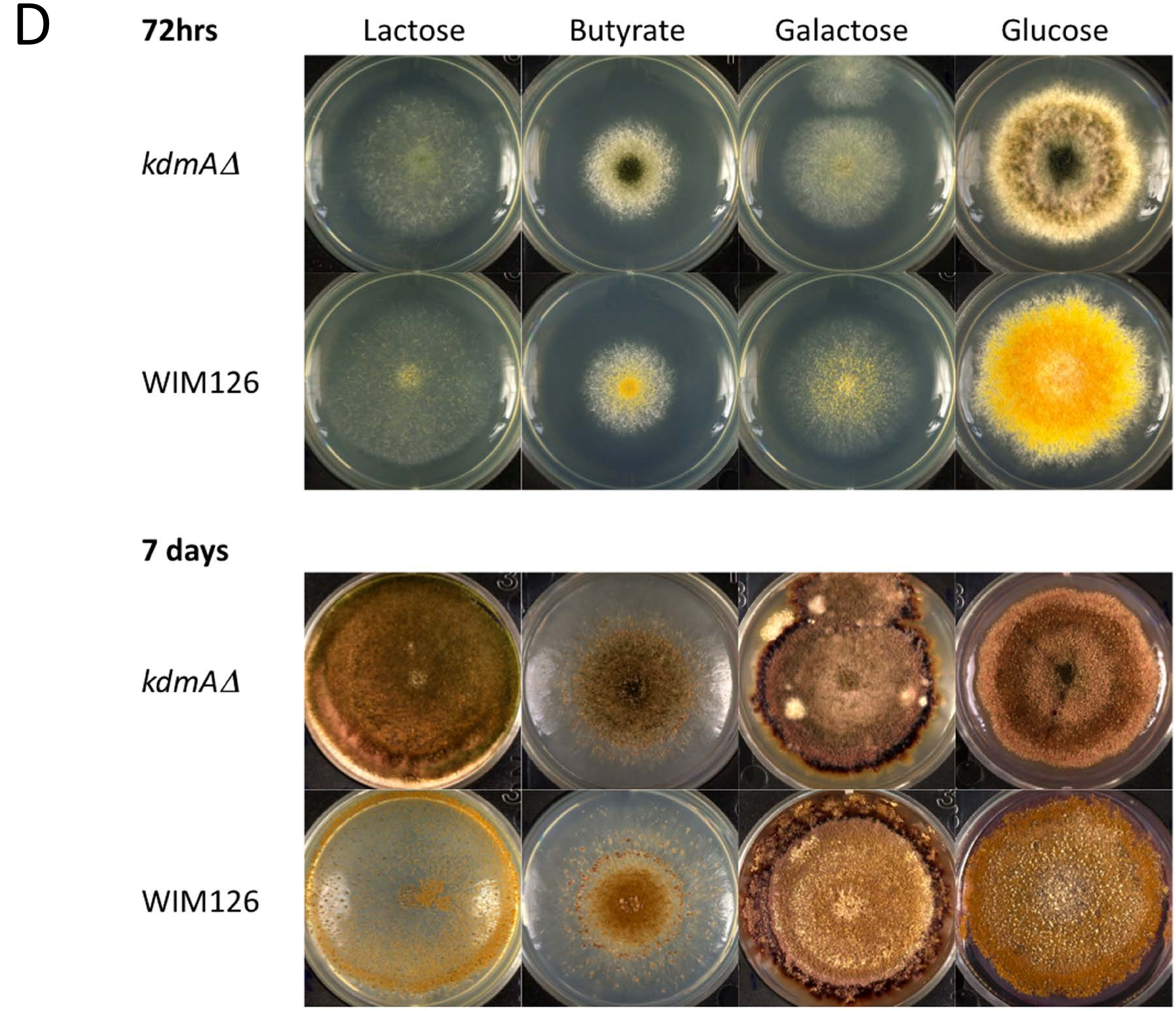
D
72hrs
Lactose
Butyrate
Galactose
Glucose
kdmAΔ
WIM126
7 days
kdmAΔ
WIM126

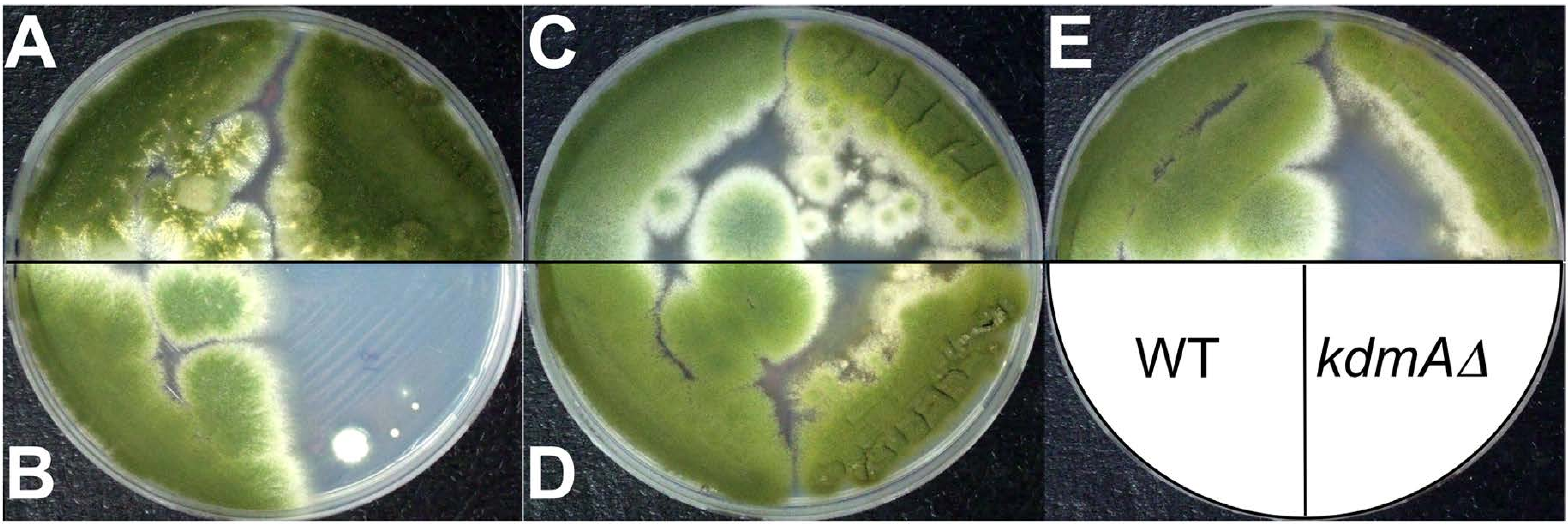

Supplementary Figure 3. Approximately 50-100 spores from WT (FGSC4) and *kdmAΔ* (DKA111) strains were streaked on complete medium and incubated at 30°C for 3 days in (**A**) constant dark or under (**B**) fluorescent white light, (**C**) red light, (**D**) blue light or (**E**) white light of 10% intensity. Plates were illuminated from above with Phillips Master TL-D 36 W/865 white fluorescent bulbs passing through a heat filter. Additional filters were used for blue-light exposure (Corning broadband blue filter with maximum transmission at 440 nm) or for red-light exposure (two Plexiglas red filters). Since these filters reduce light intensity to 10-25%, an additional control was carried out with a neutral-density filter (Schott NG4 S6) that reduces light intensity to 10% without affecting wavelength range.

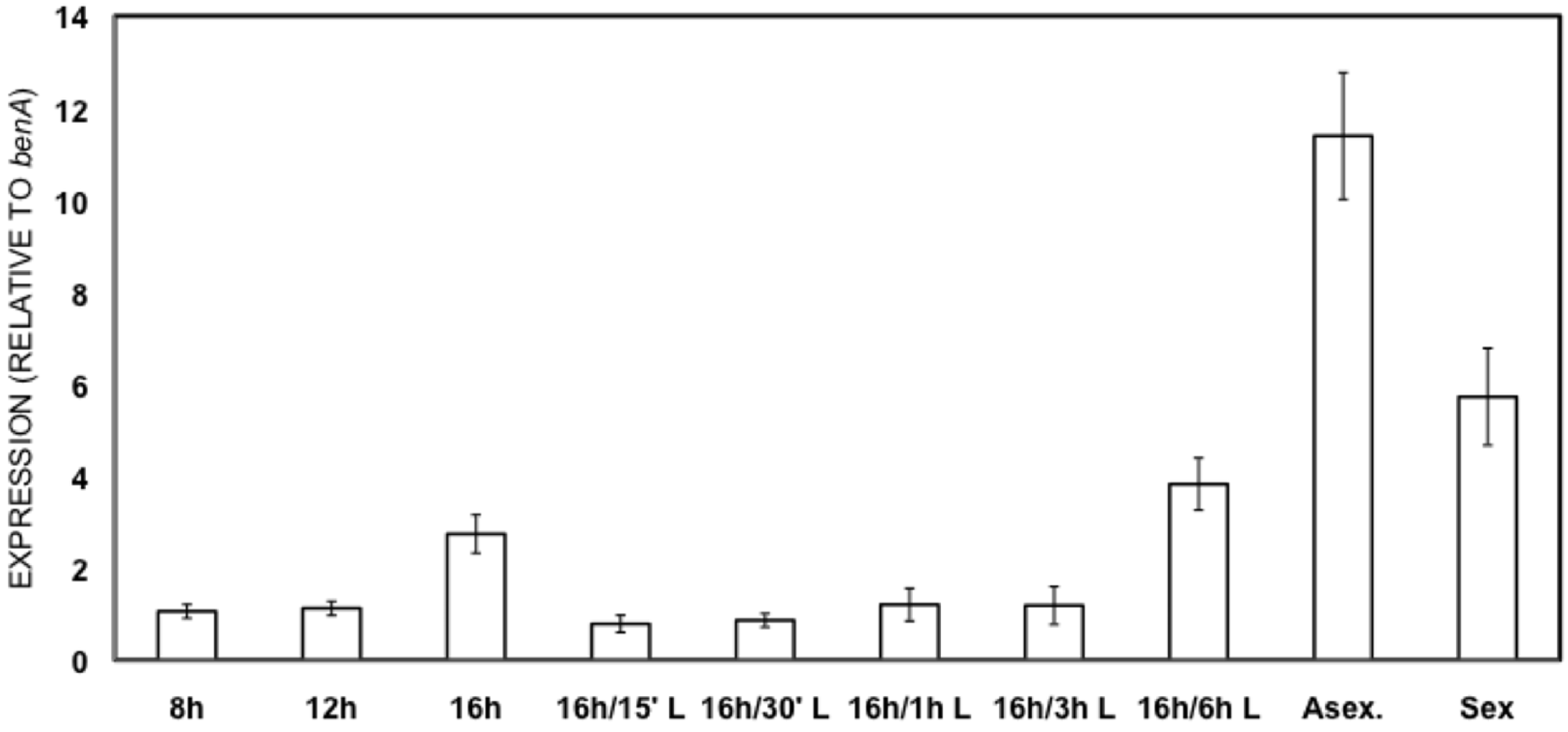

Supplementary Figure 4. *kdmA* expression in wild-type (WIM126) vegetative culture (8h, 12h, 16h), 16h vegetative cultures transferred to solid culture and exposed to light (16h/15' L, 16h/30' L, 16h/1h L, 16h/3h L, 16h/6h L) and developing samples (asexual and sexual) measured by quantitative RT-PCR, relative to tubulin *benA*. Asexual is 48 hours growth on solid culture under constant light, sexual is 72 hours in the dark, plates sealed. All samples were from complete medium. Errors are standard error of the mean from three biological replicates.

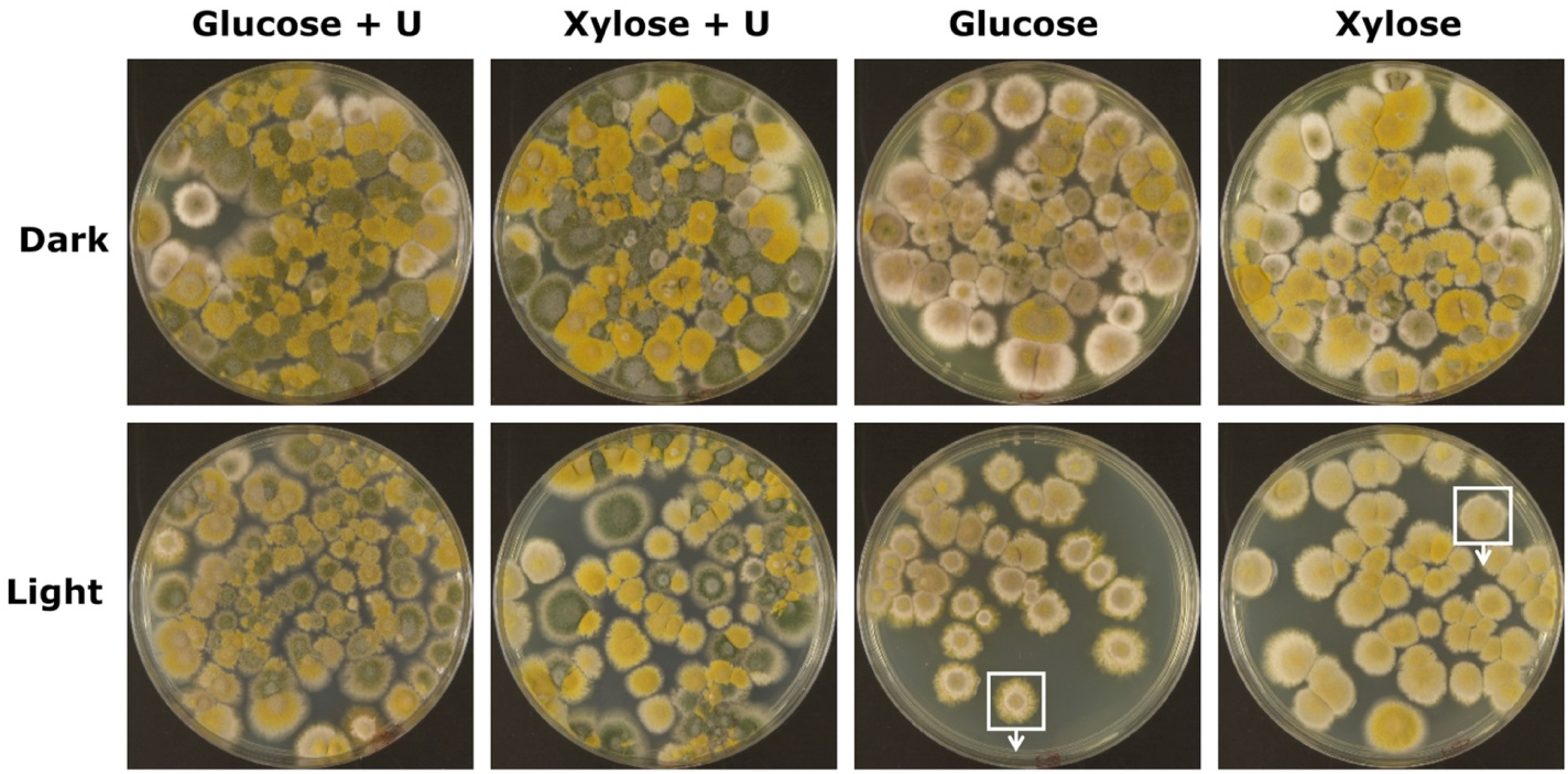

	GLUCOSE + URACIL	XYLOSE + URACIL	GLUCOSE	XYLOSE
DARK	ALL	ALL	kdmAΔ & kdmAΔ; yA::xylP(p)-kdmA	kdmAΔ & kdmAΔ; yA::xylP(p)-kdmA
LIGHT	kdmA⁺ & yA::xylP(p)-kdmA	kdmA⁺ & yA::xylP(p)-kdmA	kdmAΔ; yA::xylP(p) - kdmA	kdmAΔ; yA::xylP(p)-kdmA

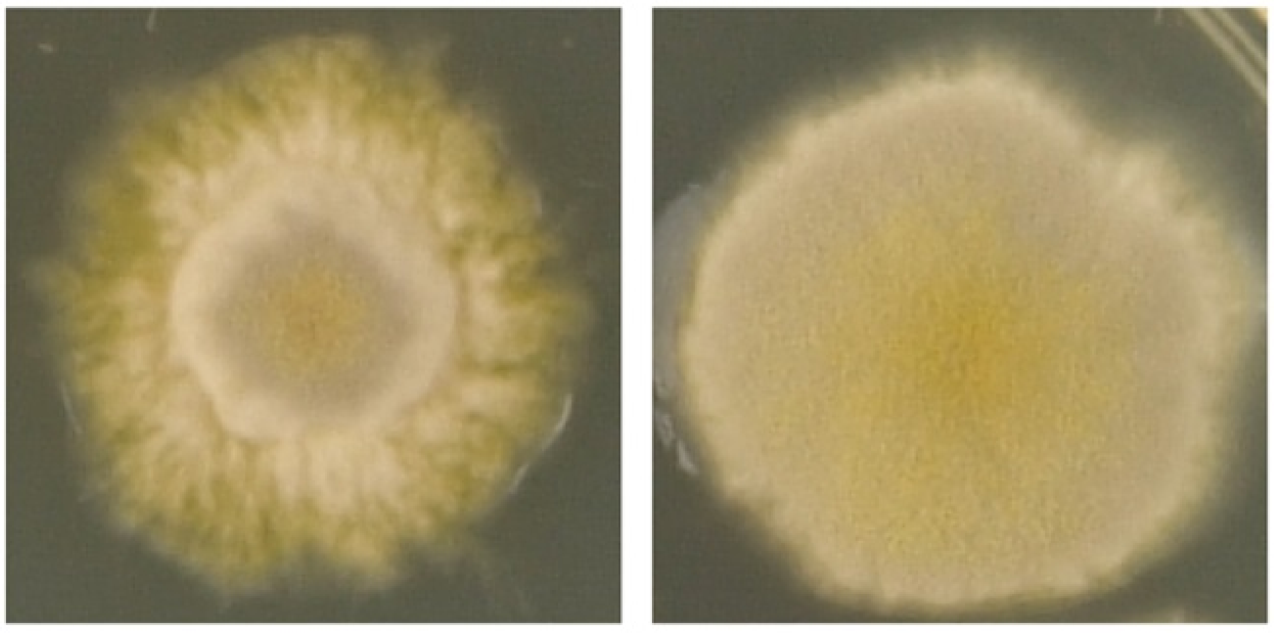

Supplementary Figure 5. ***kdmAΔ complementation and dosage sensitivity under light.*** Ascospores from a cross between *kdmAΔ* (LN12211) and *yA::xylP(p)-kdmA* (LN12289) strains were plated on complete medium with either glucose or xylose as carbon source, with or without uracil (U) supplementation, and incubated in dark or light for 2 days. Only *xylP(p)-kdmA* progeny are yellow spored due to integration at *yA* and loss of conidial laccase activity required for production of the wild-type green pigment. As summarised by corresponding table at lower left, only *kdmAΔ* progeny are able to grow without added uracil due to gene replacement with a marker conferring pyrimidine prototrophy (*N. crassa* pyr-4). Only the yellow-spored *xylP(p)-kdmA; kdmAΔ* class is able to grow under light without uracil. Without added xylose, *xylP(p)-kdmA; kdmAΔ* progeny are viable under light due to basal promoter activity, but colonies grow poorly compared with wild-type or *kdmA* induction (enlarged at lower right).

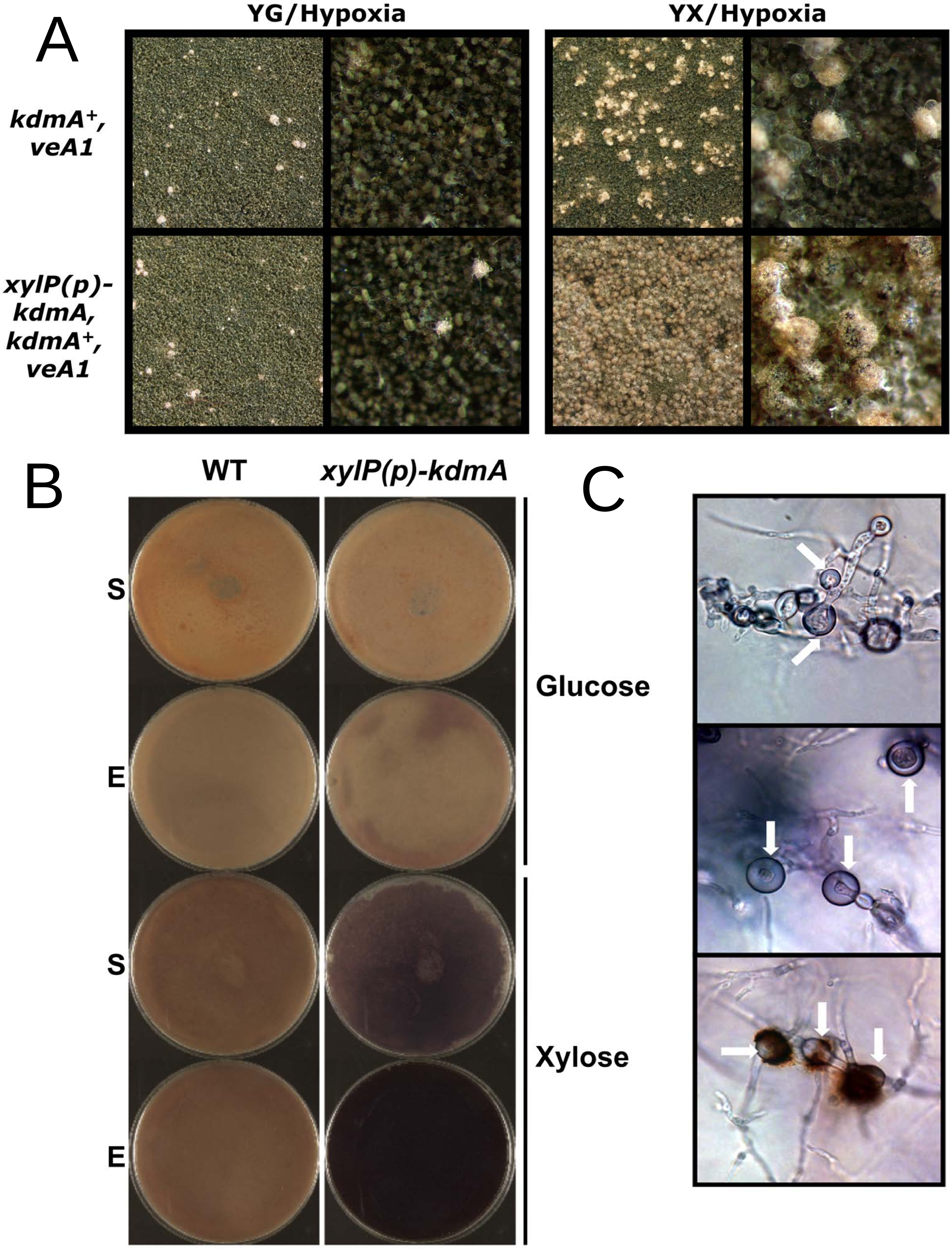

Supplementary Figure 6: *kdmA* overexpression drives sexual development in a *veA1* mutant background. Conidia from *xylP(p)-kdmA* (LN12600) and an isogenic *kdmA+* control strain (MH11037) were inoculated at a density of 103 per plate on yeast extract medium with added glucose (YG) or xylose (YX). Plates were sealed to induce hypoxia and incubated in the dark for 5 days, then unsealed and incubated for a further 1 day. Magnification is 10x (left) and 60x (right). **B**. Minimal medium containing glucose or xylose was inoculated with 10^6 ml^{-1} wild-type (LN12810) or *xylP-kdmA* (LN12809) conidia, either spread directly onto the solidified agar surface (S) or embedded (E) into molten agar to create strong hypoxia, platers were sealed and incubated in the dark for 3 days. Overexpression results in strong pigment production and production of Hulle cells (cleistothecia nurse cells) from embedded mycelia, shown enlarged in **C**. Globose Hulle cells, some associated with dense pigment agglomerations, are indicated with white arrows.

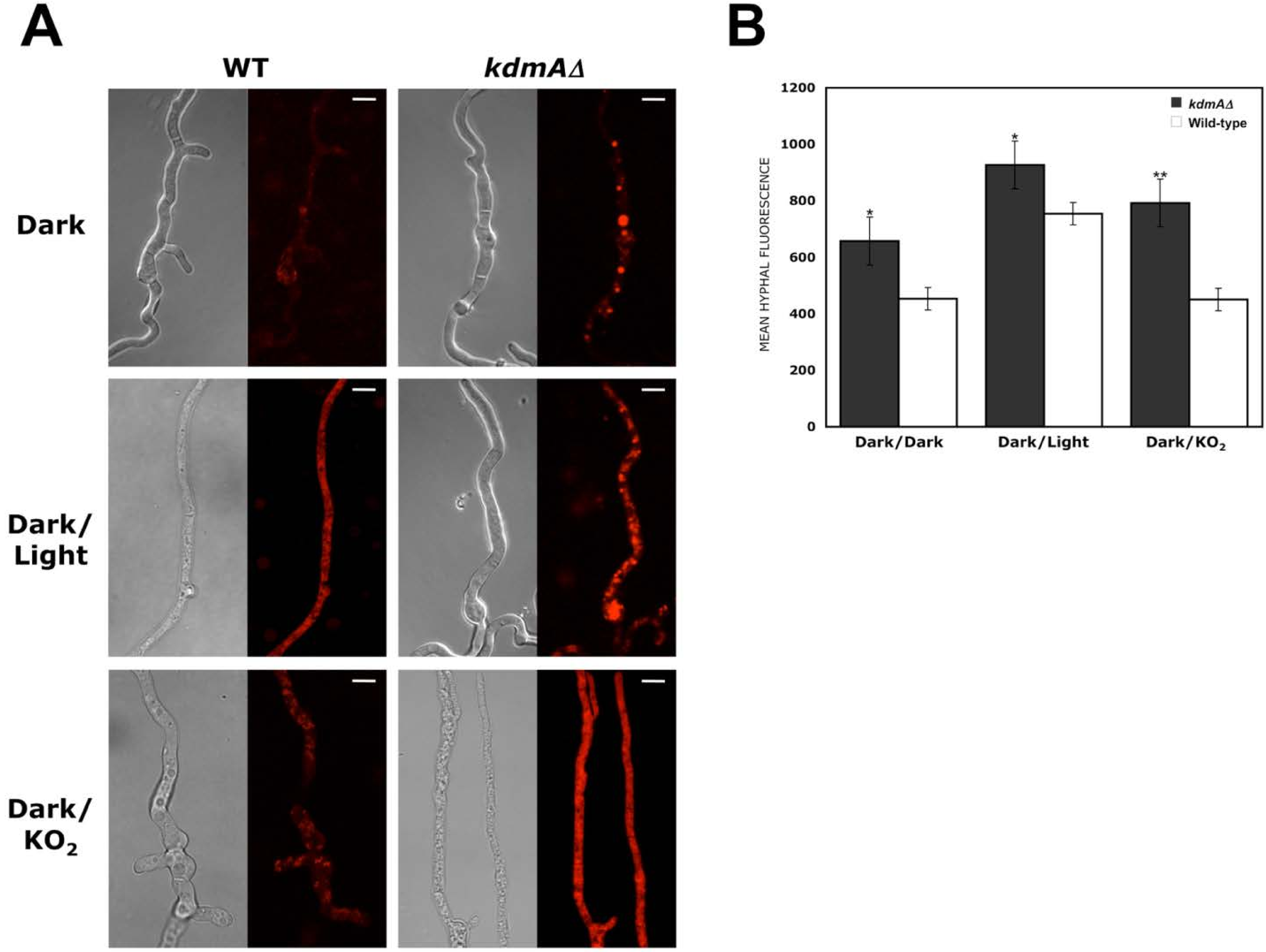

Supplementary Figure 7. Superoxide levels are increased in a *kdmA* deletion background. **A.** *kdmA*Δ (LN12211) and control strains (WIM126) were grown overnight at 25ºC, loaded with DHE for 30 minutes, followed by 30 minutes of continued dark or exposure to fluorescent white light, or treatment with 1mM potassium superoxide (KO2) in the dark. Representative images from three replicate experiments are shown. Scale bars are 5 µm. **B.** Mean oxidised DHE fluorescence for a minimum of 16 (mean of 25) images per strain per treatment. Comparisons between *kdmAΔ* and wild-type are significant by Student's t-test at $p < 0.05$ (*) and $p < 0.01$ (**). Errors are standard error.

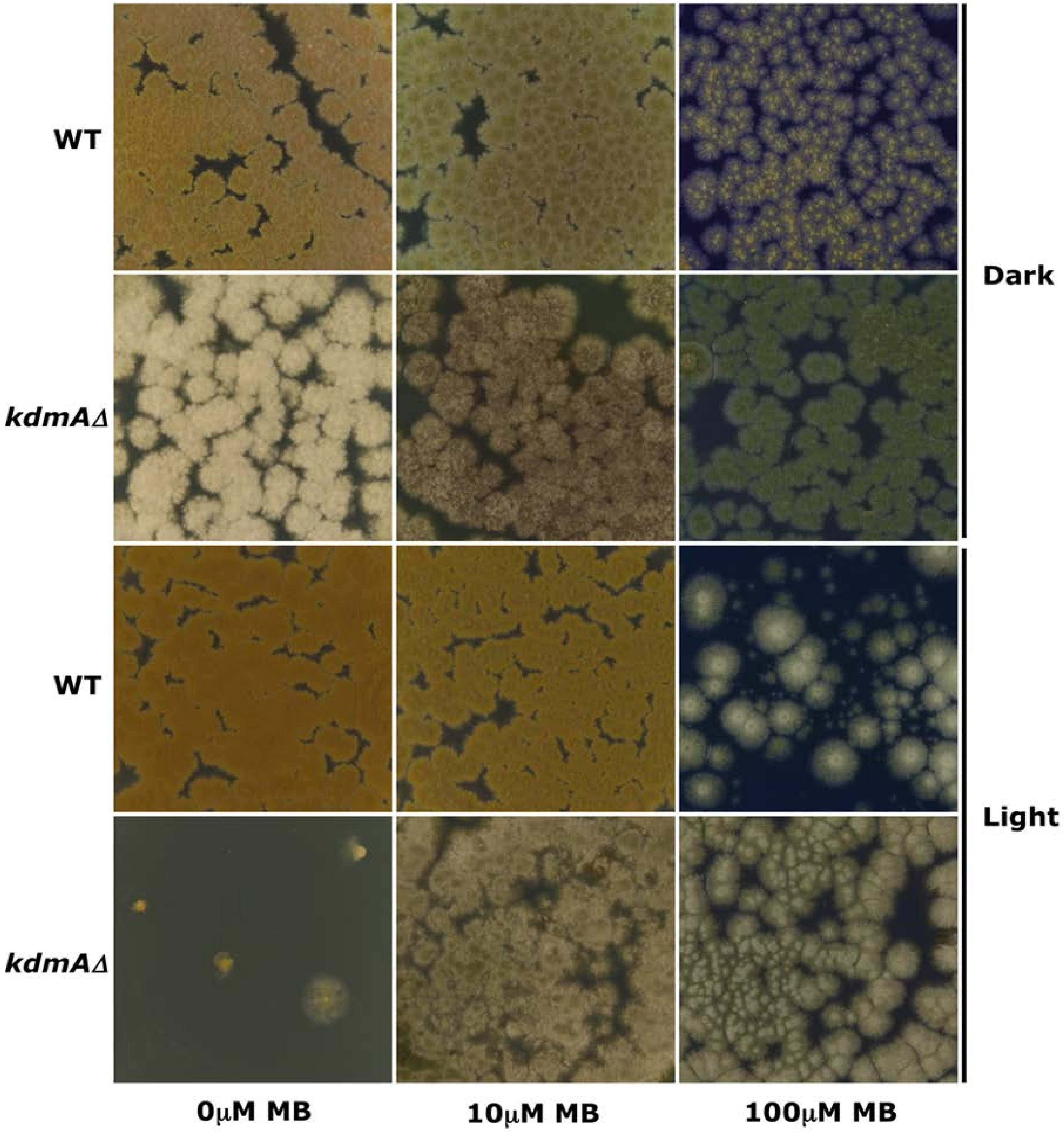

Supplementary Figure 8. *kdmAΔ* confers tolerance to the redox cycler Methylene Blue. Complete medium agar containing the photosensitive dye Methylene Blue (MB) at indicated concentrations was spread inoculated with approximately 10^3 spores of *kdmAΔ* (LN12211) and control (WIM126) strains per plate and incubated for two days in the dark or under constant white light. MB allows growth and conidiation of *kdmAΔ* under light, and the mutant is also resistant to MB-mediated photodynamic damage at 100 µM MB where the wild-type is strongly inhibited. Conversion of MB to the colourless leuco-MB form is also advanced in the mutant (compare background colour at 100 µM MB).

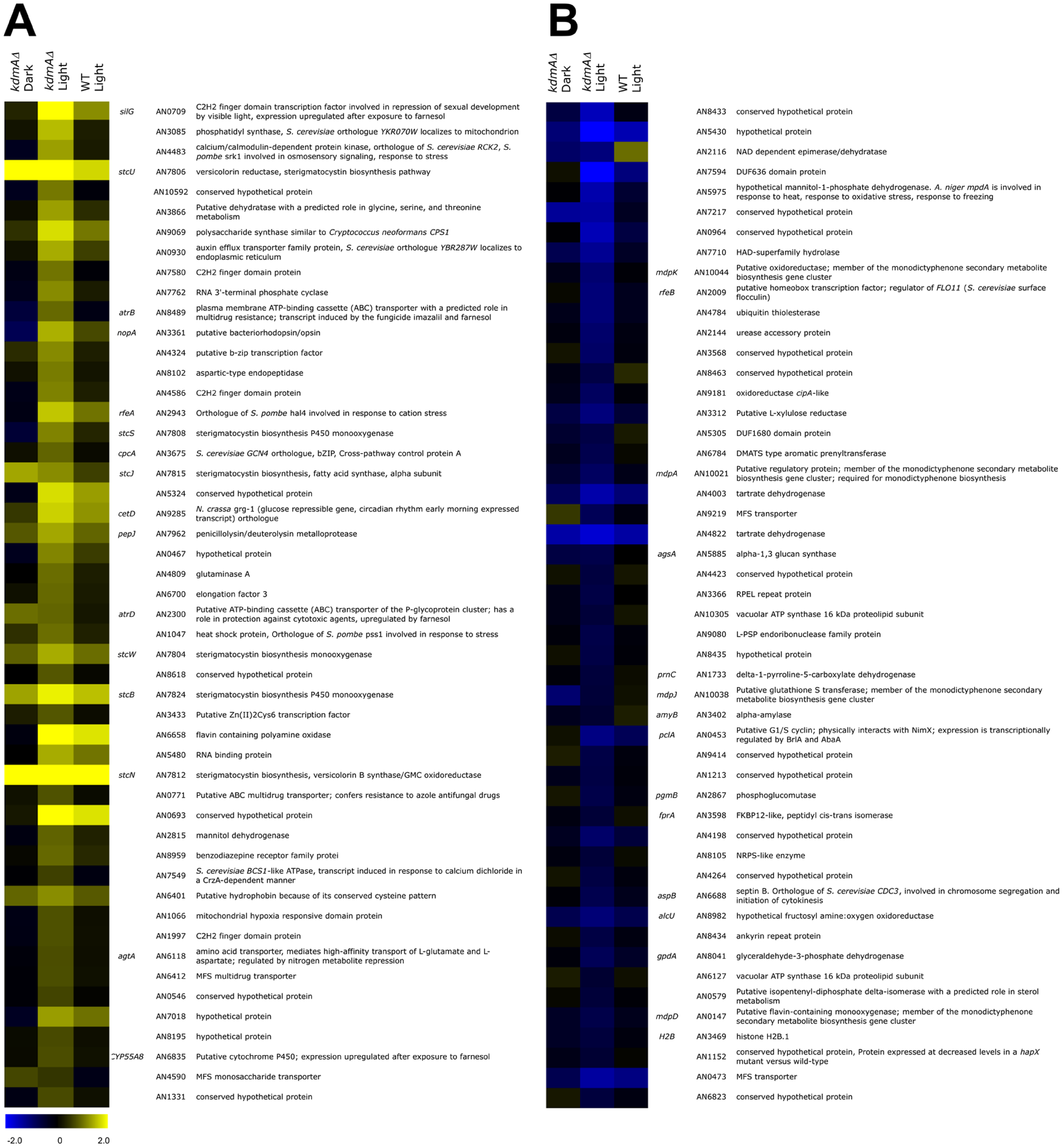

Supplementary Figure 9. Expression heat maps for genes with maximal (**A**,**C**) or minimal (**B**,**D**) expression under light and dark for *kdmAΔ* (**A**, **B**) and for both *kdmAΔ* and wild-type (**C**,**D**) under light.

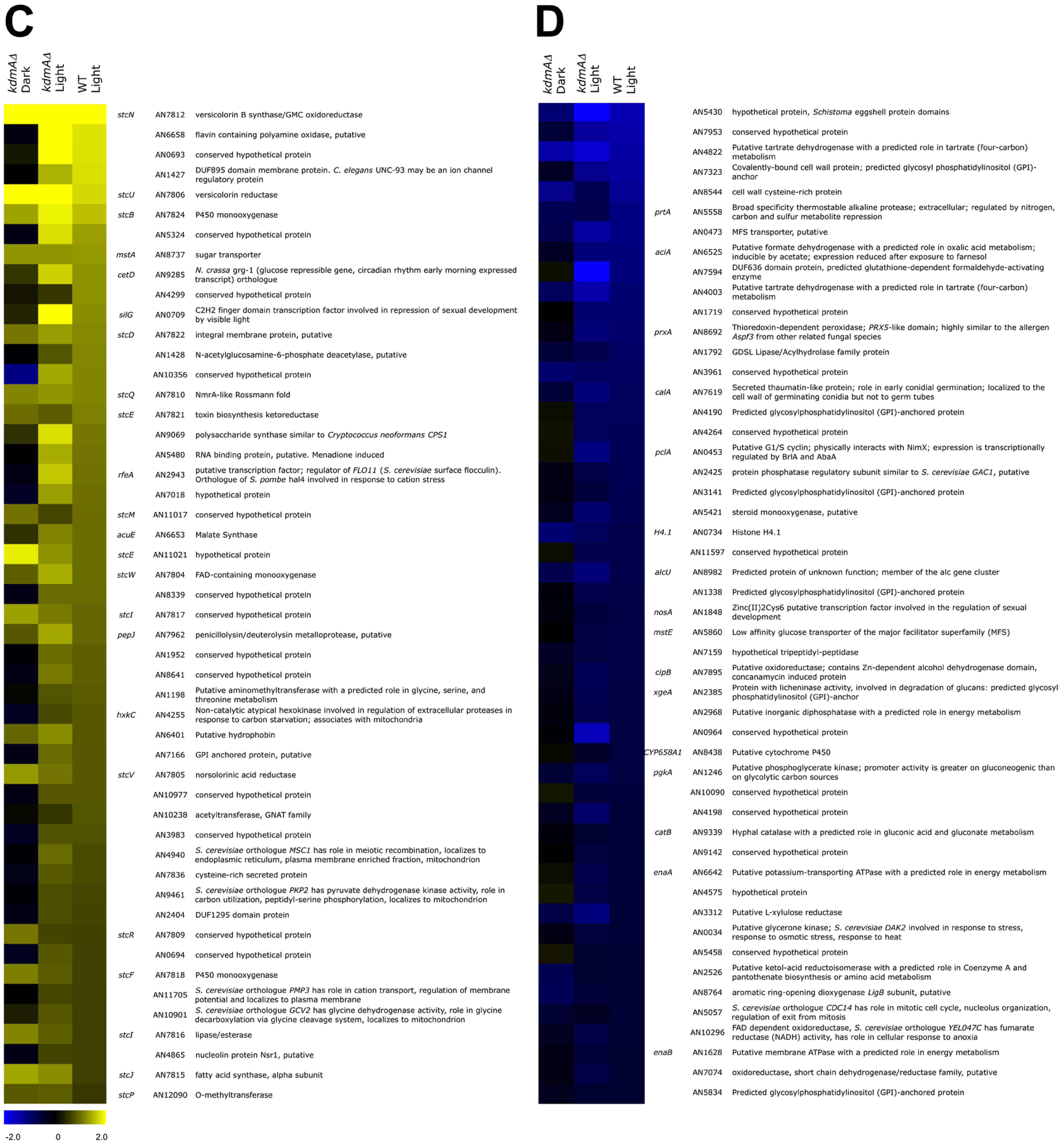
C
kdmAΔ Dark
kdmAΔ Light
WT Light
stcN AN7812 versicolorin B synthase/GMC oxidoreductase
AN6658 flavin containing polyamine oxidase, putative
AN0693 conserved hypothetical protein
AN1427 DUF895 domain membrane protein. C. elegans UNC-93 may be an ion channel regulatory protein
stcU AN7806 versicolorin reductase
stcB AN7824 P450 monooxygenase
AN5324 conserved hypothetical protein
mstA AN8737 sugar transporter
cetD AN9285 N. crassa grg-1 (glucose repressible gene, circadian rhythm early morning expressed transcript) orthologue
AN4299 conserved hypothetical protein
silG AN0709 C2H2 finger domain transcription factor involved in repression of sexual development by visible light
stcD AN7822 integral membrane protein, putative
AN1428 N-acetylglucosamine-6-phosphate deacetylase, putative
AN10356 conserved hypothetical protein
stcQ AN7810 NmrA-like Rossmann fold
stcE AN7821 toxin biosynthesis ketoreductase
AN9069 polysaccharide synthase similar to Cryptococcus neoformans CPS1
AN5480 RNA binding protein, putative. Menadione induced
rfeA AN2943 putative transcription factor; regulator of FLO11 (S. cerevisiae surface flocculin). Orthologue of S. pombe hal4 involved in response to cation stress
AN7018 hypothetical protein
stcM AN11017 conserved hypothetical protein
acuE AN6653 Malate Synthase
stcE AN11021 hypothetical protein
stcW AN7804 FAD-containing monooxygenase
AN8339 conserved hypothetical protein
stcI AN7817 conserved hypothetical protein
pepJ AN7962 penicillolysin/deuterolysin metalloprotease, putative
AN1952 conserved hypothetical protein
AN8641 conserved hypothetical protein
AN1198 Putative aminomethyltransferase with a predicted role in glycine, serine, and threonine metabolism
hxkC AN4255 Non-catalytic atypical hexokinase involved in regulation of extracellular proteases in response to carbon starvation; associates with mitochondria
AN6401 Putative hydrophobin
AN7166 GPI anchored protein, putative
stcV AN7805 norsolorinic acid reductase
AN10977 conserved hypothetical protein
AN10238 acetyltransferase, GNAT family
AN3983 conserved hypothetical protein
AN4940 S. cerevisiae orthologue MSC1 has role in meiotic recombination, localizes to endoplasmic reticulum, plasma membrane enriched fraction, mitochondrion
AN7836 cysteine-rich secreted protein
AN9461 S. cerevisiae orthologue PKP2 has pyruvate dehydrogenase kinase activity, role in carbon utilization, peptidyl-serine phosphorylation, localizes to mitochondrion
AN2404 DUF1295 domain protein
stcR AN7809 conserved hypothetical protein
AN0694 conserved hypothetical protein
stcF AN7818 P450 monooxygenase
AN11705 S. cerevisiae orthologue PMP3 has role in cation transport, regulation of membrane potential and localizes to plasma membrane
AN10901 S. cerevisiae orthologue GCV2 has glycine dehydrogenase activity, role in glycine decarboxylation via glycine cleavage system, localizes to mitochondrion
stcI AN7816 lipase/esterase
AN4865 nucleolin protein Nsr1, putative
stcJ AN7815 fatty acid synthase, alpha subunit
stcP AN12090 O-methyltransferase
-2.0
0
2.0
D
kdmAΔ Dark
kdmAΔ Light
WT Light
AN5430 hypothetical protein, Schistoma eggshell protein domains
AN7953 conserved hypothetical protein
AN4822 Putative tartrate dehydrogenase with a predicted role in tartrate (four-carbon) metabolism
AN7323 Covalently-bound cell wall protein; predicted glycosyl phosphatidylinositol (GPI)-anchor
AN8544 cell wall cysteine-rich protein
prtA AN5558 Broad specificity thermostable alkaline protease; extracellular; regulated by nitrogen, carbon and sulfur metabolite repression
AN0473 MFS transporter, putative
aciA AN6525 Putative formate dehydrogenase with a predicted role in oxalic acid metabolism; inducible by acetate; expression reduced after exposure to farnesol
AN7594 DUF636 domain protein, predicted glutathione-dependent formaldehyde-activating enzyme
AN4003 Putative tartrate dehydrogenase with a predicted role in tartrate (four-carbon) metabolism
AN1719 conserved hypothetical protein
prxA AN8692 Thioredoxin-dependent peroxidase; PRX5-like domain; highly similar to the allergen Aspf3 from other related fungal species
AN1792 GDSL Lipase/Acylhydrolase family protein
AN3961 conserved hypothetical protein
calA AN7619 Secreted thaumatin-like protein; role in early conidial germination; localized to the cell wall of germinating conidia but not to germ tubes
AN4190 Predicted glycosylphosphatidylinositol (GPI)-anchored protein
AN4264 conserved hypothetical protein
pclA AN0453 Putative G1/S cyclin; physically interacts with NimX; expression is transcriptionally regulated by BrlA and AbaA
AN2425 protein phosphatase regulatory subunit similar to S. cerevisiae GAC1, putative
AN3141 Predicted glycosylphosphatidylinositol (GPI)-anchored protein
AN5421 steroid monooxygenase, putative
H4.1 AN0734 Histone H4.1
AN11597 conserved hypothetical protein
alcU AN8982 Predicted protein of unknown function; member of the alc gene cluster
AN1338 Predicted glycosylphosphatidylinositol (GPI)-anchored protein
nosA AN1848 Zinc(II)2Cys6 putative transcription factor involved in the regulation of sexual development
mstE AN5860 Low affinity glucose transporter of the major facilitator superfamily (MFS)
AN7159 hypothetical tripeptidyl-peptidase
cipB AN7895 Putative oxidoreductase; contains Zn-dependent alcohol dehydrogenase domain, concanamycin induced protein
xgeA AN2385 Protein with licheninase activity, involved in degradation of glucans: predicted glycosyl phosphatidylinositol (GPI)-anchor
AN2968 Putative inorganic diphosphatase with a predicted role in energy metabolism
AN0964 conserved hypothetical protein
CYP658A1 AN8438 Putative cytochrome P450
pgkA AN1246 Putative phosphoglycerate kinase; promoter activity is greater on gluconeogenic than on glycolytic carbon sources
AN10090 conserved hypothetical protein
AN4198 conserved hypothetical protein
catB AN9339 Hyphal catalase with a predicted role in gluconic acid and gluconate metabolism
AN9142 conserved hypothetical protein
enaA AN6642 Putative potassium-transporting ATPase with a predicted role in energy metabolism
AN4575 hypothetical protein
AN3312 Putative L-xylulose reductase
AN0034 Putative glycerone kinase; S. cerevisiae DAK2 involved in response to stress, response to osmotic stress, response to heat
AN5458 conserved hypothetical protein
AN2526 Putative ketol-acid reductoisomerase with a predicted role in Coenzyme A and pantothenate biosynthesis or amino acid metabolism
AN8764 aromatic ring-opening dioxygenase LigB subunit, putative
AN5057 S. cerevisiae orthologue CDC14 has role in mitotic cell cycle, nucleolus organization, regulation of exit from mitosis
AN10296 FAD dependent oxidoreductase, S. cerevisiae orthologue YEL047C has fumarate reductase (NADH) activity, has role in cellular response to anoxia
enaB AN1628 Putative membrane ATPase with a predicted role in energy metabolism
AN7074 oxidoreductase, short chain dehydrogenase/reductase family, putative
AN5834 Predicted glycosylphosphatidylinositol (GPI)-anchored protein

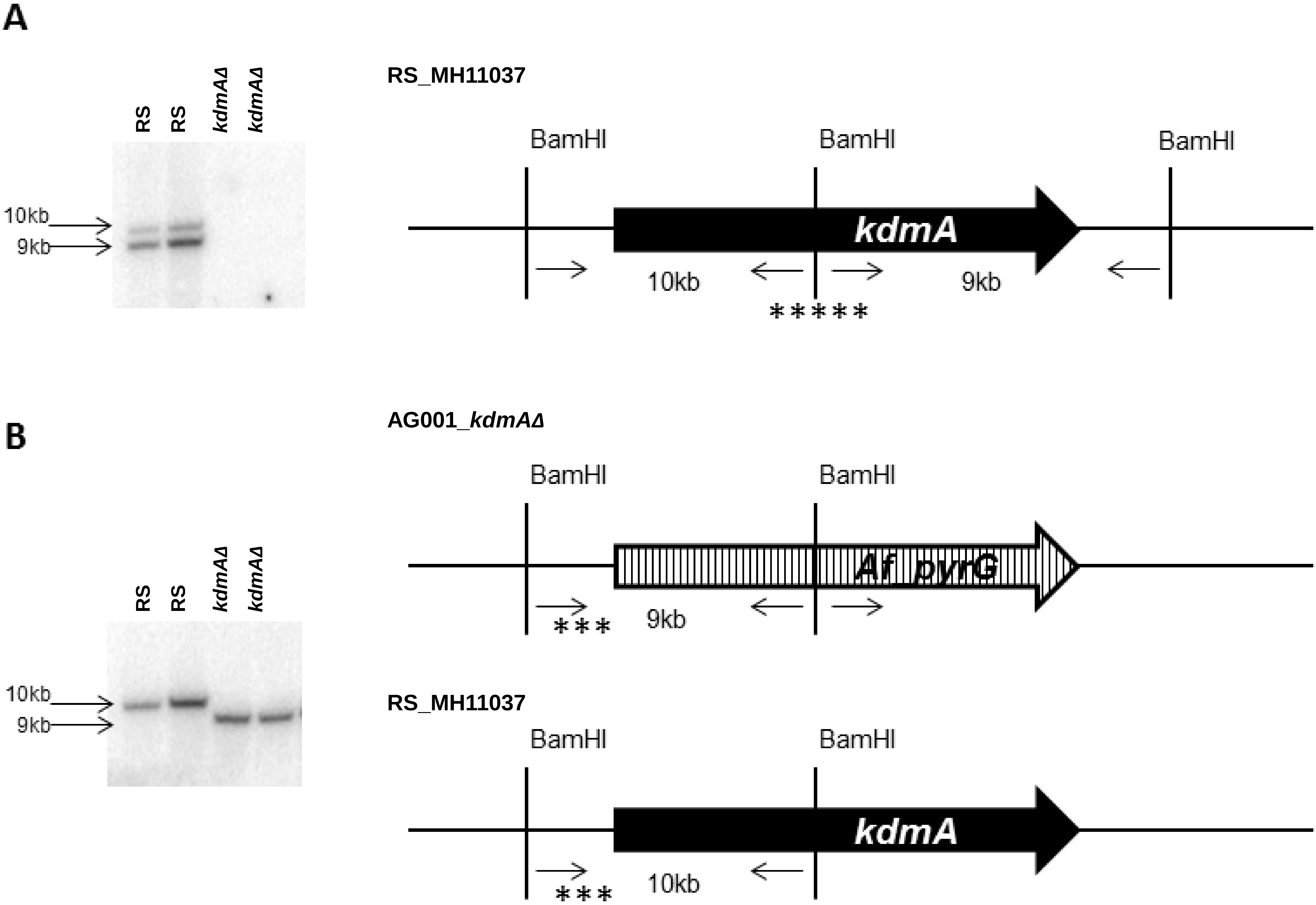

Supplementary Figure 10. Southern blot confirming deletion of *kdmA* ORF (**A**) and correct upstream integration (**B**). Black and dashed arrows represent ORFs, asterisks indicate Southern blot probe binding sites. RS- recipient strain MH11037 (TNO2A25).

Strain ID	Genotype	Source
MH11037	*pyrG89; pabaB22 nkuAΔ(argB); veA1 riboB2*	M. J. Hynes (unpublished)
WIM126	*yA2 pabaA1*	L. N. Yager
LN12063	*pyrG89; pabaB22 nkuAΔ(argB); veA1 kdmAΔ(Nc pyr-4) riboB2*	This study
LN12211	*pabaA1 pyrG89; kdmAΔ(Ncr pyr-4)*	This study
LN12289	*yA::[pLN7807] pyrG89; pyroA4; veA1*	This study
LO1946	*pyrG89; pyroA4; hhoA-GFP(Afu pyroA); veA1 riboB2*	Oakley Lab (Kansas University)
LN12465	*pyrG89; hhoA-GFP(Afu pyroA); kdmAΔ(Nc pyr-4)*	This study
LN12508	*pabaA1; hhoA-GFP(Afu pyroA)*	This study
LN12600	*pabaA1 pyrG89; kdmA[pLN7807]*	This study
LN12809	*yA2 pabaA1 pyrG89; kdmA::[pLN7807]*	This study
LN12810	*yA2 pabaA1 pyrG89*	This study
LN12042	*yA2 pabaA1 pyrG89; pyroA4 nkuAΔ(Bar)*	This study
LN12923	*nkuAΔ(Bar); veA1 sodMΔ(Af pyrG) riboB2*	This study
LN12927	*pabaA1, pyroA4 nkuA(Bar); fhdAΔ(Af pyrG)*	This study
LN12974	*fhbAΔ(Af pyrG) pyroA4 nkuA(Bar); veA1*	This study
LN12955	*srrAΔ(Af pyrG); pyroA4 nkuA(Bar); veA1*	This study
YS10542	*biA1; pabaB22; msnAΔ(Af pyrG); veA1 niiA4*	Y. Suzuki (unpublished)
MH12650	*nkuA(argB); AN2099Δ(Af pyroA); veA1 niiA4*	M.J. Hynes (unpublished)
CLK14	*biA1; catAΔ(argB); metG1; catBΔ(argB) veA1*	Kawasaki *et al.* (1997)
LN12945	*pabaA1; pyroA4 nkuA(Bar); AN9060Δ(Af pyrG)*	This study
AG001	*pabaB22, riboB2; nkuAΔ(argB), kdmAΔ(Af pyrG), veA1*	This study
AG004	*pabaB22; nkuAΔ(argB), kdmAΔ(Af pyrG), veA1*	This study
WT	*pabaA1, veA1*	Pontecorvo *et al.* , (1953)
YR1108	*clrDΔ::pyrG, argB2 pyro yA2 veA1*	Reyes-Dominguez *et al.*, (2010)
DKA111	*pabaA1; kdmAΔ(Nc pyr-4)*	This study

Supplementary Table 1. *A. nidulans* strains

Name	Oligonucleotide
AN5999pF	TCAGTCTTGGGCGTGCGGCAGAA
AN5999pR	CATTGCGGCGCGCACTATTTGAC
AN5999 ORF F	TCACATCGAGTCTGCAACCAAGGAG
AN5999 ORF R	TTACAGAAGCTTGCCGCCAACGAAG
AN6000p-220F	CCAAAAGTCCCCGTATGGC
AN6000p-1R	TGTTACTTCTCTGCTGGTCG
AN6000 ORF F	ACTAACCCGAGTCGCTGAGA
AN6000 ORF R	TATCATTCACCATGCCGAGA
aflR RT F	AGCCCAGCTGGTGCTGAGCGAGCTATAC
aflR RT R	CCAGGGTGGTCGACGACAAGGGGGT
benA ChIP F	GATGGCTGCCTCTGACTTCCG
benA ChIP R	GCGCATCTGGTCCTCAACCTC
ipnA RT F	GGAGACGACCAAGCAGCCAAA
ipnA RT R	TTTTCCCGGGGATGGACAGG
AN1060 UP F	GATCAGCTTTTAACCCCTATCAAAACC
AN1060 UP R	CGCTATGAAGAGTATGGAAAAGAG
AN1060 UPNESTED F	GAATCGAGCTAGCTAGCCGACATGC
AN1060_PYRG F	TTGAACAGCTTTCATTCTCTTTTCCATACTCTTCATAGCGGAATTCGCCTCAAACAATGCTC
AN1060_PYRG R	GATGATGCAGTTACAGCGGCATGTCTATAAGCAGTCATAGGAATTCTCAGTCCTGCTCC
AN1060_DOWN F	CTATGACTGCTTATAGACATGCCGCTG
AN1060_DOWN R	ATCGGCCATACTCAACAGCACAACC
AN1060_DOWNNESTED R	CCACAGCGAAATCGCACTCAATATC
AN1060_ORF F	TGGATTGACGTGGAAGAGATCGAGCG

Supplementary Table 2. Oligonucleotides
